# Mechanochemical Strategies Applied to the Late‐Stage Modifications of Pharmaceutically Active Compounds

**DOI:** 10.1002/anie.202503061

**Published:** 2025-08-28

**Authors:** Johanna Templ, Lars Borchardt

**Affiliations:** ^1^ Inorganic Chemistry I Ruhr‐Universität Bochum Universitätsstraße 150 44801 Bochum Germany

**Keywords:** Ball milling, Green chemistry, Medicinal mechanochemistry, Solvent‐free synthesis, Sustainable drug design

## Abstract

This review explores the potential of mechanochemistry in the late‐stage modification of active pharmaceutical ingredients (APIs), offering a comprehensive analysis of methods designed to transform structurally complex molecular scaffolds by examining the scope, efficiency, and mechanistic aspects of these approaches. To further assist researchers, we provide a detailed table summarizing the discussed APIs, their respective modifications, and any necessary prefunctionalizations. This resource should provide a practical guide for selecting suitable substrates to evaluate the pharmaceutical relevance of existing and novel (mechano)chemical methods.

## Graphical abstract

This review highlights mechanochemical strategies for the late‐stage modification of active pharmaceutical ingredients (APIs), providing a valuable resource for selecting substrates to evaluate the pharmaceutical potential of novel and existing (mechano)chemical protocols.

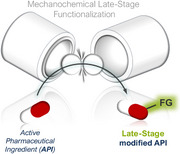

## 1 Introduction

In recent years, mechanochemistry has gained increasing attention as a transformative approach within synthetic chemistry, leveraging mechanical energy to promote chemical transformations.^[^ ^1^, ^2^, ^3^ ^]^ By circumventing reliance on bulk solvents, increasing energy efficiency and enabling unique reaction pathways, mechanochemistry aligns perfectly with the principles of green chemistry.^[^ ^4^, ^5^ ^]^ Its potential to address some of the most pressing challenges in modern chemical synthesis, including waste reduction, operational simplicity, and energy efficiency, has made it a valuable tool in the pursuit of sustainable practices across various industries.^[^ ^6^, ^7^, ^8^ ^]^ Nowhere is this more evident than in the pharmaceutical and fine chemical sectors, which face increasing pressure to adopt environmentally friendly methodologies in the face of heightened environmental awareness, especially in terms of solvent waste reduction.

Although mechanochemistry has found widespread application in the synthesis of small molecules, intermediates, and polymers,^[^ ^9^, ^10^, ^11^ ^]^ its role in the late‐stage functionalization of active pharmaceutical ingredients (APIs) is only beginning to be explored. Late‐stage modifications occupy a critical niche in drug discovery and development. These transformations, often carried out on structurally complex molecules, enable precise alterations to pharmacologically relevant frameworks, fine‐tuning their biological properties such as potency, selectivity, metabolic stability, and solubility.^[^ ^12^, ^13^, ^14^, ^15^ ^]^ Moreover, late‐stage modifications provide a route for generating analogues from existing scaffolds, facilitating structure–activity relationship (SAR) studies.^[^ ^16^, ^17^ ^]^

In this context, mechanochemistry offers distinct advantages over traditional solution‐based methods. Its ability to perform transformations under solvent‐free conditions drastically reduces solvent waste, simplifies purification, and minimizes the environmental footprint of chemical processes. In times of growing environmental awareness, the application of mechanochemical methods will become increasingly important not only for the synthesis, but also for the late‐stage functionalization (LSF) of APIs.^[^ ^18^, ^19^, ^20^, ^21^, ^22^ ^]^ Despite the growing body of research in this area, a review specifically focusing on mechanochemical strategies applied in late‐stage API functionalization remains absent. Current literature reviews have largely focused on the mechanosynthesis of APIs or intermediates thereof, overlooking the remarkable potential of mechanochemical strategies that have proven effective for transforming structurally complex compounds, such as APIs.^[^ ^19^, ^20^, ^23^, ^24^, ^25^, ^26^ ^]^ These overlooked strategies hold significant promise for pharmaceutical synthesis and modification but have yet to receive adequate attention.

This review seeks to bridge this gap by providing a comprehensive overview of mechanochemical protocols specifically applied to the late‐stage modification of marketed APIs, emphasizing their practical utility and transformative potential for pharmaceutical development. By systematically categorizing current methodologies based on bond‐forming reactions, we aim to offer a clear and organized, but also critical, perspective on the field. The review will cover five main categories of transformations: C─C bond formation, C─N bond formation, C─O bond formation, C─X bond formation, and miscellaneous reactions. Each section will detail scope, efficiency, mechanistic findings, and challenges of methods that have demonstrated success in modifying marketed drugs or bioactive compounds.

Beyond an extensive discussion of mechanochemical protocols, this review includes two dedicated sections. The first examines the advantages of mechanochemical processes over solution‐based methods, particularly in the context of pharmaceutical applications. The second addresses current limitations and key challenges that hinder the full implementation of mechanochemistry in drug discovery and synthesis, alongside potential strategies and emerging technologies that could help overcome these barriers in the future. Finally, a comprehensive tabular summary detailing all pharmaceutically active compounds discussed, their respective late‐stage functionalization, and any necessary prefunctionalization is provided (Table 1). Organized alphabetically by compound, this overview is designed to serve as a quick reference for researchers seeking suitable substrates for testing new (mechanochemical) methods or evaluating the pharmaceutical relevance of established protocols. Additionally, a schematic overview of the mechanochemical devices used in the discussed syntheses is provided in the final section of this review.

**Table 1:** Overview of APIs or bioactive compounds discussed in this review, organized alphabetically, along with their respective mechanochemical late‐stage functionalizations by research group and any necessary prefunctionalization of the API or bioactive compound.

**Entry**	**Original** API or bioactive compound	**Modified** API or bioactive compound by group	Reaction type and prefunctionalization of API
1	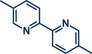 **Abametapir**	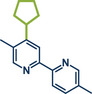 **29** – Yu 2023^[^ ^27^ ^]^	**Late‐Stage modification**: Radical C(*sp^2^*)‐H alkylation **Prefunctionalization of API**: None
2	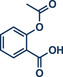 **Aspirin**	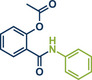 **47** – Kulkarni 2023^[^ ^28^ ^]^	**Late‐Stage modification**: EDC coupling amidation **Prefunctionalization of API**: None
3	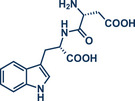 **Asp‐Trp dipeptide**	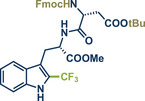 **13** – Ito 2020^[^ ^29^ ^]^	**Late‐Stage modification**: Radical trifluoromethylation **Prefunctionalization of API**: Fmoc‐protection and esterification
4	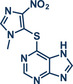 **Azathioprine**	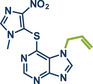 **40** – Templ and Schnürch 2024^[^ ^30^ ^]^	**Late‐Stage modification**: Tsuji–Trost allylation **Prefunctionalization of API**: None
5	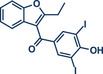 **Benziodarone**	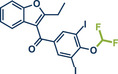 **61** – Gouverneur 2023^[^ ^31^ ^]^	**Late‐Stage modification**: Difluoromethylation **Prefunctionalization of API**: None
6	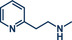 **Betahistine**	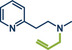 **41** – Templ and Schnürch 2024^[^ ^30^ ^]^	**Late‐Stage modification**: Tsuji–Trost allylation **Prefunctionalization of API**: None
7	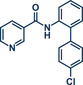 **Boscalid**	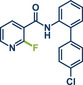 **67** – Kubota and Ito 2025^[^ ^32^ ^]^	**Late‐Stage modification**: Nucleophilic aromatic fluorination **Prefunctionalization of API**: None
8	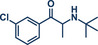 **Bupropion**	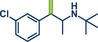 **38** – Templ and Schnürch 2024^[^ ^33^ ^]^	**Late‐Stage modification**: Wittig olefination **Prefunctionalization of API**: None
9	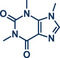 **Caffeine**	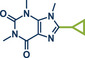 **31** – Yu and Su 2024^[^ ^34^ ^]^	**Late‐Stage modification**: Radical Minisci C(*sp^2^*)‐H alkylation **Prefunctionalization of API**: None
10	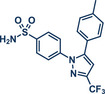 **Celecoxib**	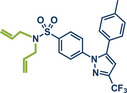 **45** – Templ and Schnürch 2024^[^ ^30^ ^]^	**Late‐Stage modification**: Tsuji–Trost allylation **Prefunctionalization of API**: None
11	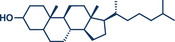 **Cholestanol/Coprostanol**	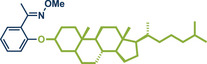 **55** – Lou and Xu 2021^[^ ^35^ ^]^	**Late‐Stage modification**: C(*sp^2^*)‐O coupling **Prefunctionalization of API**: None
12	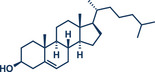 **Cholesterol**	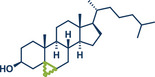 **24b** – Browne 2023^[^ ^36^ ^]^	**Late‐Stage modification**: Simmons–Smith Cyclopropanation **Prefunctionalization of API**: None
13		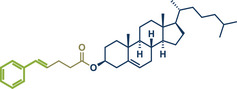 **24a** – Yu 2024^[^ ^37^ ^]^	**Late‐Stage modification**: Oxidative Heck coupling **Prefunctionalization of API**: Esterification for alkene attachment
14	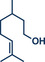 **Citronellol**	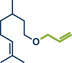 **58** – Templ and Schnürch 2024^[^ ^30^ ^]^	**Late‐Stage modification**: Tsuji‐Trost allylation **Prefunctionalization of API**: None
15	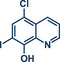 **Clioquinol**	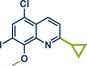 **33** – Yu and Su 2024^[^ ^34^ ^]^	**Late‐Stage modification**: Radical Minisci C(*sp^2^*)‐H alkylation **Prefunctionalization of API**: Methylation of hydroxy group
16	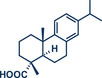 **Dehydroabietic acid**	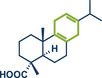 **73** – Ito 2023^[^ ^38^ ^]^	**Late‐Stage modification**: Birch reduction **Prefunctionalization of API**: None
17	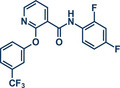 **Diflufenican**	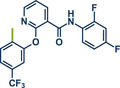 **7a** – Pilarski 2021^[^ ^39^ ^]^	**Late‐Stage modification**: C(*sp^2^*)‐H methylation **Prefunctionalization of API**: None
18		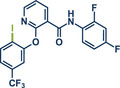 **7b** – Pilarski 2024^[^ ^40^ ^]^	**Late‐Stage modification**: C(*sp^2^*)‐H iodination **Prefunctionalization of API**: None
19	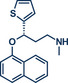 **Duloxetine**	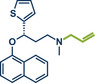 **42** – Templ and Schnürch 2024^[^ ^30^ ^]^	**Late‐Stage modification**: Tsuji‐Trost allylation **Prefunctionalization of API**: None
20	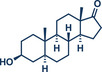 **Epiandrosterone**	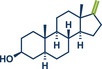 **37** – Templ and Schnürch 2024^[^ ^33^ ^]^	**Late‐Stage modification**: Wittig olefination **Prefunctionalization of API**: None
21	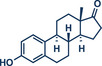 **Estrone**	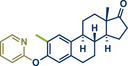 **8a** – Pilarski 2021^[^ ^39^ ^]^	**Late‐Stage modification**: C(*sp^2^*)‐H methylation **Prefunctionalization of API**: Pyridine directing group attachment
22		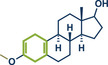 **8c** – Ito 2023^[^ ^38^ ^]^	**Late‐Stage modification**: Birch reduction **Prefunctionalization of API**: Methylation of the hydroxy group
23		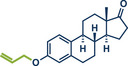 **8b** – Templ and Schnürch 2024^[^ ^30^ ^]^	**Late‐Stage modification**: Tsuji‐Trost allylation **Prefunctionalization of API**: None
24	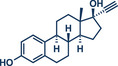 **Ethynylestradiol**	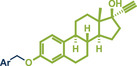 **63a** – Bolm 2024^[^ ^41^ ^]^	**Late‐Stage modification**: Mitsunobu Reaction **Prefunctionalization of API**: None
25		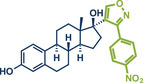 **63b** – Hernandez 2024^[^ ^42^ ^]^	**Late‐Stage modification**: 1,3‐Dipolar cycloaddition **Prefunctionalization of API**: None
26	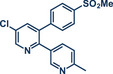 **Etoricoxib**	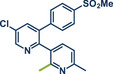 **5a** – Pilarski 2021^[^ ^39^ ^]^	**Late‐Stage modification**: C(*sp^2^*)‐H methylation **Prefunctionalization of API**: None
27		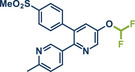 **5b** – Gouverneur 2023^[^ ^31^ ^]^	**Late‐Stage modification**: Difluoromethylation **Prefunctionalization of API**: Hydroxylation of the C(*sp^2^*)─Cl bond
28	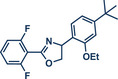 **Etoxazole**	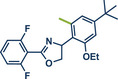 **2a –** Pilarski 2021 and 2023^[^ ^39^, ^43^ ^]^	**Late‐Stage modification**: C(*sp^2^*)‐H methylation **Prefunctionalization of API**: None
29		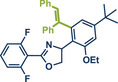 **2b** – Pilarski 2024^[^ ^40^ ^]^	**Late‐Stage modification**: C(*sp^2^*)‐H alkenylation **Prefunctionalization of API**: None
30	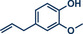 **Eugenol**	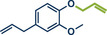 **57** – Templ and Schnürch 2024^[^ ^30^ ^]^	**Late‐Stage modification**: Tsuji‐Trost allylation **Prefunctionalization of API**: None
31	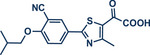 **Fenbuxostat**	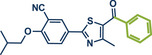 **15** – Szostak 2022^[^ ^44^ ^]^	**Late‐Stage modification**: Suzuki–Miyaura coupling via C─N bond cleavage **Prefunctionalization of API**: Conversion of acid into active *N*‐acyl‐glutarimide
32	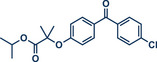 **Fenofibrate**	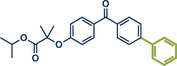 **9a** – Pilarski 2024^[^ ^40^ ^]^	**Late‐Stage modification**: Suzuki–Miyaura coupling **Prefunctionalization of API**: None
33		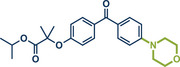 **9b** – Pilarski 2024^[^ ^40^ ^]^	**Late‐Stage modification**: Buchwald–Hartwig coupling **Prefunctionalization of API**: None
34		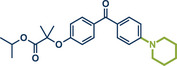 **9c** – Rueping 2024^[^ ^45^ ^]^	**Late‐Stage modification**: Ni‐catalyzed C(*sp^2^*)‐Cl cross‐coupling amination **Prefunctionalization of API**: None
35	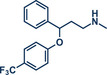 **Fluoxetine**	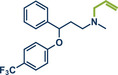 **43** – Templ and Schnürch 2024^[^ ^30^ ^]^	**Late‐Stage modification**: Tsuji–Trost allylation **Prefunctionalization of API**: None
36	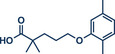 **Gemfibrozil**	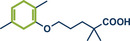 **23b** – Ito 2024^[^ ^38^ ^]^	**Late‐Stage modification**: Birch reduction **Prefunctionalization of API**: None
37		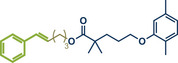 **23a** – Yu 2024^[^ ^37^ ^]^	**Late‐Stage modification**: Oxidative Heck coupling **Prefunctionalization of API**: Esterification for alkene attachment
38	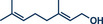 **Geraniol**	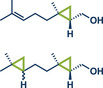 **25a, 25b** – Browne 2023^[^ ^36^ ^]^	**Late‐Stage modification**: Simmons–Smith cyclopropanation **Prefunctionalization of API**: None
39	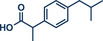 **Ibuprofen**	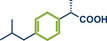 **22d** – Ito 2024^[^ ^38^ ^]^	**Late‐Stage modification**: Birch reduction **Prefunctionalization of API**: None
40		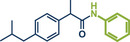 **22b** – Kulkarni 2023^[^ ^28^ ^]^	**Late‐Stage modification**: EDC coupling amidation **Prefunctionalization of API**: None
41		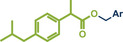 **22c** – Bolm 2024^[^ ^41^ ^]^	**Late‐Stage modification**: Mitsunobu reaction **Prefunctionalization of API**: None
42		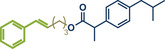 **22a** – Yu 2024^[^ ^37^ ^]^	**Late‐Stage modification**: Oxidative Heck coupling **Prefunctionalization of API**: Esterification for alkene attachment
43	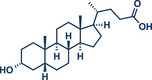 **Lithocholic acid**	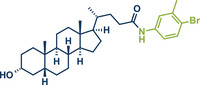 **50** – Aav and Kananovich 2024^[^ ^46^ ^]^	**Late‐Stage modification**: EDC coupling amidation **Prefunctionalization of API**: None
44	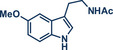 **Melatonin**	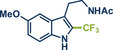 **12** – Ito 2020^[^ ^29^ ^]^	**Late‐Stage modification**: Radical trifluoromethylation **Prefunctionalization of API**: None
45	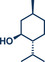 **Menthol**	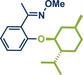 **28b** – Lou and Xu 2021^[^ ^35^ ^]^	**Late‐Stage modification**: C(*sp^2^*)‐O coupling **Prefunctionalization of API**: None
46		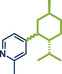 **28a** – Yu 2023^[^ ^27^ ^]^	**Late‐Stage modification**: Radical C(*sp^2^*)‐H alkylation **Prefunctionalization of API**: Conversion of hydroxy group to bromide
47	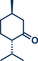 **Menthone**	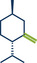 **36** – Templ and Schnürch 2024^[^ ^33^ ^]^	**Late‐Stage modification**: Wittig olefination **Prefunctionalization of API**: None
48	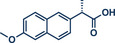 **Naproxen**	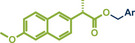 **62** – Bolm 2024^[^ ^41^ ^]^	**Late‐Stage modification**: Mitsunobu reaction **Prefunctionalization of API**: None
49	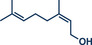 **Nerol**	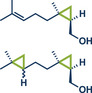 **26a, 26b** – Browne 2023^[^ ^36^ ^]^	**Late‐Stage modification**: Simmons–Smith cyclopropanation **Prefunctionalization of API**: None
50	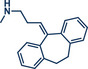 **Nortriptyline**	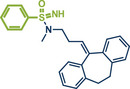 **70** – Bolm 2024^[^ ^47^ ^]^	**Late‐Stage modification**: Oxidative amination **Prefunctionalization of API**: None
51	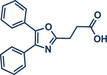 **Oxaprozin**	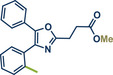 **1a** – Pilarski 2021 and 2023^[^ ^39^, ^43^ ^]^	**Late‐Stage modification**: C(*sp^2^*)‐H methylation **Prefunctionalization of API**: Esterification
52		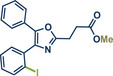 **1c** – Pilarski 2024^[^ ^40^ ^]^	**Late‐Stage modification**: C(*sp^2^*)‐H iodination **Prefunctionalization of API**: Esterification
53		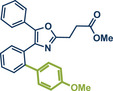 **1b** – Pilarski 2024^[^ ^40^ ^]^	**Late‐Stage modification**: Suzuki–Miyaura coupling **Prefunctionalization of API**: Esterification and iodination (see entry 52)
54	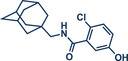 **P2X7R ligand**	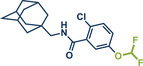 **59** – Gouverneur 2023^[^ ^31^ ^]^	**Late‐Stage modification**: Difluoromethylation **Prefunctionalization of API**: None
55	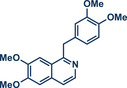 **Papaverine**	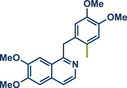 **3** – Pilarski 2021 and 2023^[^ ^39^, ^43^ ^]^	**Late‐Stage modification**: C(*sp^2^*)‐H methylation **Prefunctionalization of API**: None
56	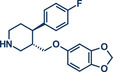 **Paroxetine**	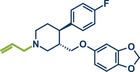 **44** – Templ and Schnürch 2024^[^ ^30^ ^]^	**Late‐Stage modification**: Tsuji–Trost allylation **Prefunctionalization of API**: None
57	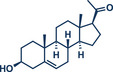 **Pregnenolone**	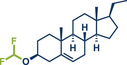 **46b** – Bolm 2023^[^ ^48^ ^]^	**Late‐Stage modification**: Difluoromethylation **Prefunctionalization of API**: Ketone reduction to alkene
58		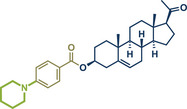 **46a** – Rueping 2024^[^ ^45^ ^]^	**Late‐Stage modification**: Ni‐catalyzed C(*sp^2^*)‐Cl cross‐coupling amination **Prefunctionalization of API**: Esterification with *p*‐bromobenzoic acid
59	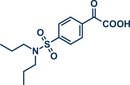 **Probenecid**	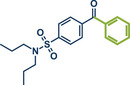 **16a** – Szostak 2022^[^ ^44^ ^]^	**Late‐Stage modification**: Suzuki–Miyaura coupling via C─N bond cleavage **Prefunctionalization of API**: Conversion of acid into active *N*‐acyl‐glutarimide
60		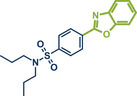 **16b** – Szostak 2024^[^ ^49^ ^]^	**Late‐Stage modification**: C(*sp^2^*)‐C(*sp^2^*) decarboxylative cross‐coupling **Prefunctionalization of API**: Conversion of acid into active *N*‐acyl‐glutarimide
61	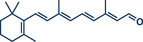 **Retinal**	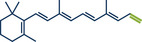 **39** – Templ and Schnürch 2024^[^ ^33^ ^]^	**Late‐Stage modification**: Wittig olefination **Prefunctionalization of API**: None
62	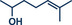 **Sulcatol**	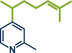 **27** – Yu 2023^[^ ^27^ ^]^	**Late‐Stage modification**: Radical C(*sp^2^*)‐H alkylation **Prefunctionalization of API**: Conversion of hydroxy group to bromide
63	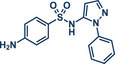 **Sulfaphenazole**	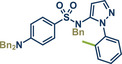 **6** – Pilarski 2021^[^ ^39^ ^]^	**Late‐Stage modification**: C(*sp^2^*)‐H methylation **Prefunctionalization of API**: *N*‐Benzylation
64	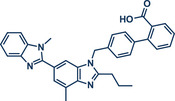 **Telemisartan**	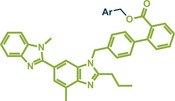 **64** – Bolm 2024^[^ ^41^ ^]^	**Late‐Stage modification**: Mitsunobu reaction **Prefunctionalization of API**: None
65	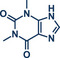 **Theophylline**	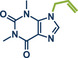 **32b** – Templ and Schnürch 2024^[^ ^30^ ^]^	**Late‐Stage modification**: Tsuji–Trost allylation **Prefunctionalization of API**: None
66		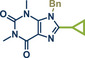 **32a** – Yu and Su 2024^[^ ^34^ ^]^	**Late‐Stage modification**: Radical Minisci C(*sp^2^*)‐H alkylation **Prefunctionalization of API**: *N*‐Benzylation
67	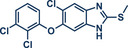 **Triclabendazole**	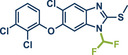 **60** – Gouverneur 2023^[^ ^31^ ^]^	**Late‐Stage modification**: Difluoromethylation **Prefunctionalization of API**: None
68	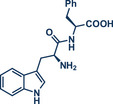 **Trp‐Phe dipeptide**	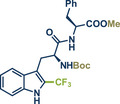 **14** – Ito 2020^[^ ^29^ ^]^	**Late‐Stage modification**: Radical trifluoromethylation **Prefunctionalization of API**: Boc‐protection and esterification
69	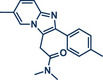 **Zolpidem**	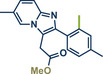 **4** – Pilarski 2023^[^ ^43^ ^]^	**Late‐Stage modification**: C(*sp^2^*)‐H methylation **Prefunctionalization of API**: Ester formation

## 2 C─C Bond Forming Reactions

In late‐stage functionalization of bioactive compounds, the methyl group—the smallest alkyl group—often exerts profound effects on pharmacological properties compared to longer alkyl chains. Adding a single methyl group can influence bioavailability, potency, metabolic stability, and binding affinity, collectively known as the “magic methyl effect”.^[^ ^50^, ^51^, ^52^ ^]^ However, achieving selective methylation at this stage poses significant challenges, including the prevention of overalkylation and issues with chemo‐ and regioselectivity.^[^ ^14^, ^17^, ^53^, ^54^ ^]^ These challenges are particularly pronounced in late‐stage C─H activation, where nonfunctionalized C─H bonds are targeted using transition metal catalysts. For complex drug molecules, the presence of multiple C─H bonds complicates precise regiocontrol.^[^ ^17^ ^]^ The use of directing groups—preferably traceless—has emerged as a critical strategy to address these issues.^[^ ^40^, ^55^, ^56^ ^]^

**Figure 1:**
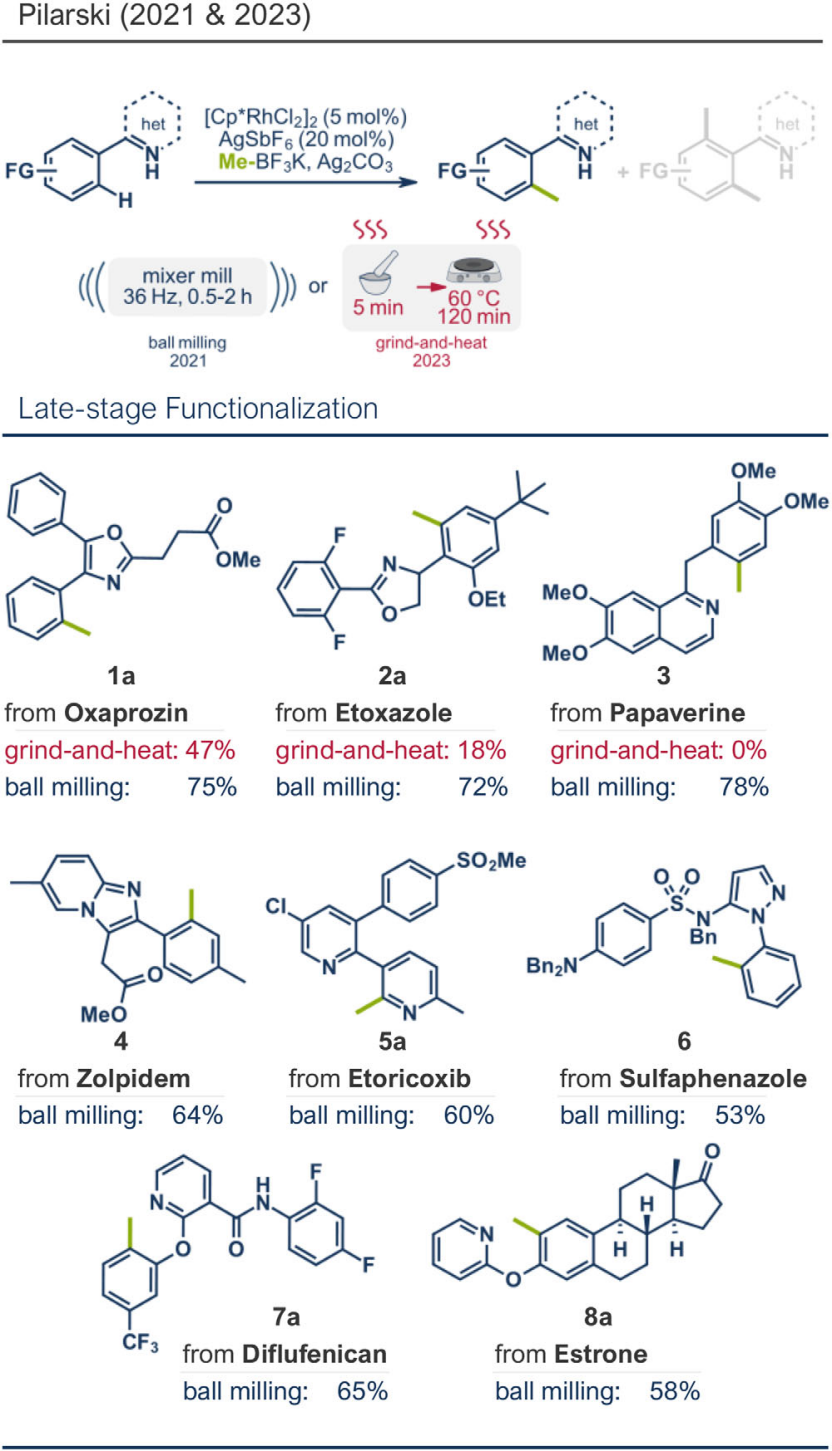
Grind‐and‐heat protocol for a directing group mediated C(*sp^2^*)‐H activation/methylation by the group of Pilarski. Adapted with permission.^[^ ^39^ ^]^ © 2023 Royal Society of Chemistry.

In 2021, the Pilarski group disclosed a solvent‐free, mechanochemical approach combining directing‐group‐mediated C─H methylation with ball milling (Figure 1).^[^ ^39^ ^]^ Using 2‐phenylpyridine as a model substrate, the heterocyclic nitrogen directed [Cp*RhCl_2_]_2_ to selectively activate the *ortho*‐position of the phenyl ring via 5‐ or 6‐membered rhodacycles. Depending on the substrate, either Me‐B(OH)_2_ or MeBF_3_K was employed as a methylating agent, avoiding toxic reagents such as MeI or SnMe_4_. The protocol required Ag_2_CO_3_ as an additive, with AgSbF_6_ (20 mol%) additionally needed for reactions involving 6‐membered rhodacycles. The solvent‐free method demonstrated superior regioselectivity, achieving mono‐to‐difunctionalized product ratios of up to 32:1, compared to 3–4:1 in solution. Reaction times under mechanochemical conditions were reduced to a maximum of 2 h (cf. 16–24 h in solution), and yields were often higher. The protocol exhibited excellent functional group tolerance, enabling late‐stage methylation of various bioactive compounds and derivatives, including etoxazole (pesticide, product **2a**), papaverine (antispasmodic, product **3**), etoricoxib (NSAID, product **5a**), estrone (hormone, product **8a**), sulfaphenazole (antibacterial, product **6**), and diflufenican (herbicide, product **7a**). Two years later, the same group reported a similar C─H activation/methylation protocol under “grind‐and–heat” conditions.^[^ ^43^ ^]^ In this approach, reagents were manually ground with a mortar and pestle for 5 min, followed by heating at 60 °C for 2 h. Although the grind‐and‐heat method simplified operations by obviating specialized ball‐milling equipment, it often produced significantly lower yields in late‐stage methylation of bioactive molecules. Some substrates showed no conversion at all (cf. product **3**). These results suggest that certain reactions, such as C─H activation/methylation, may require the efficient and continuous mixing provided by ball milling, with the mechanical impact and shear forces inherent to this technique aiding rapid and high yielding functionalization under solvent‐free conditions.

**Figure 2:**
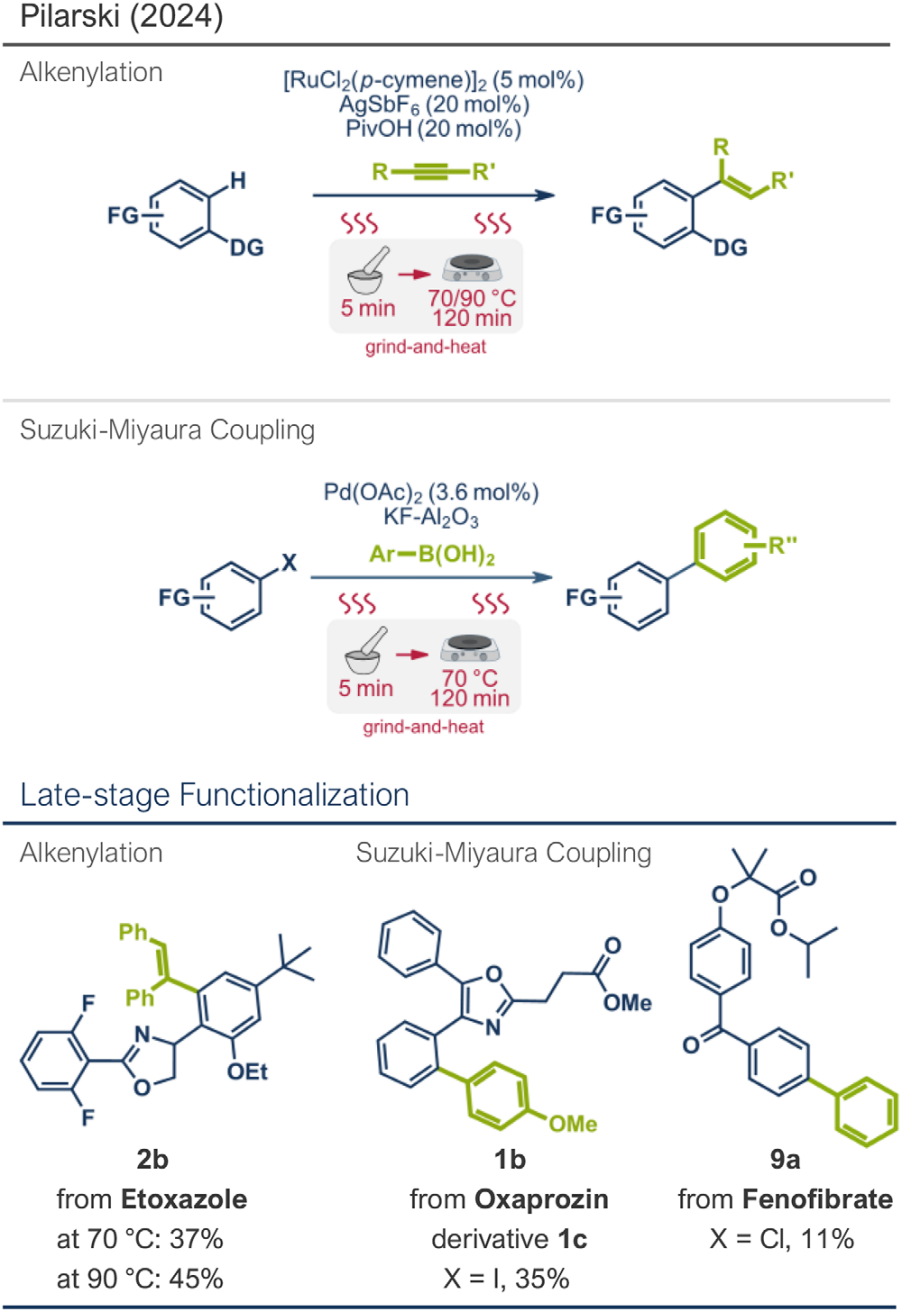
Grind‐and‐heat protocol for a directing group mediated alkenylation and a Suzuki–Miyaura coupling by the group of Pilarski. Adapted with permission.^[^ ^40^ ^]^ © 2024 Preprint – Cambridge Open Engage (CC BY‐NC‐ND 4.0).

In 2024, the Pilarski group explored the general applicability of grind‐and‐heat processes as an alternative to ball milling approaches, adapting several established protocols from the literature and often expanding the substrate scope to include structurally complex bioactive compounds (Figure 2).^[^ ^40^ ^]^ One notable example is the Ru‐catalyzed C─H alkenylation of directing‐group‐containing substrates using alkynes, adapted from prior literature,^[^ ^57^, ^58^ ^]^ which gave C─H‐modified etoxazole **2b** in 37% yield using grind‐and‐heat methods.^[^ ^40^ ^]^ Additionally, Pilarski's group achieved the functionalization of fenofibrate, an antilipemic drug, via a palladium‐catalyzed Suzuki–Miyaura cross‐coupling targeting its C(*sp^2^*)─Cl bond. The conditions for this transformation were adapted from an electromagnetic milling protocol developed by Liu and coworkers.^[^ ^59^ ^]^ Applying the same conditions in a grind‐and‐heat setup, the team achieved a late‐stage arylation of fenofibrate, albeit with an 11% yield (product **9a**). Additionally, they employed this method to further functionalized modified oxaprozin derivative **1c**, converting it into its anisole‐containing analogue **1b**.^[^ ^40^ ^]^

**Figure 3:**
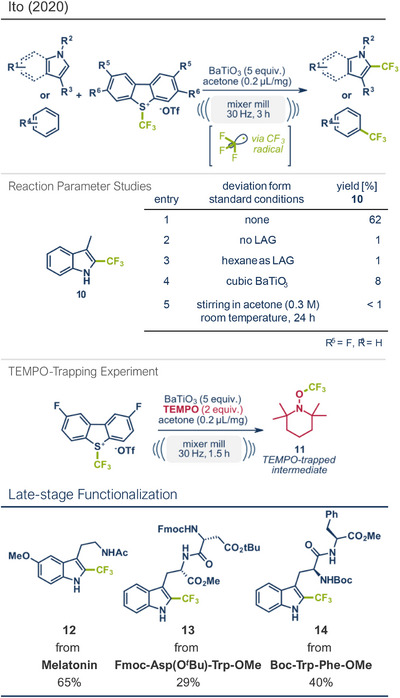
Radical trifluoromethylation protocol via mechanochemical activation in a ball milling reactor by the group of Ito. Adapted with permission.^[^ ^29^ ^]^ © 2020 Wiley‐VCH GmbH.

In addition to methylation, trifluoromethylation has emerged as a pivotal strategy in the late‐stage modification of drug molecules. Fluorine substituents often enhance a drug's bioavailability, protein interactions, and metabolic stability, making their incorporation a valuable tool in medicinal chemistry.^[^ ^60^, ^61^, ^62^ ^]^ Thus solvent‐free, mechanochemical fluorination strategies suitable for drug functionalization are highly desirable, potentially paving the way for greener pharmaceutical syntheses. Driven by this demand, Kubota, Ito and coworkers investigated mechanoredox systems suitable for the C‐H trifluoromethylation of aromatic compounds (Figure 3).^[^ ^29^ ^]^ Their work builds upon a pioneering report by the same group a year earlier, which demonstrated the potential of piezoelectric materials (e.g., BaTiO_3_) to induce redox reactions under mechanical stress from ball milling, offering an alternative to photoredox catalysis.^[^ ^63^ ^]^ In the trifluoromethylation protocol, the mechanical agitation of the ball mill polarized BaTiO_3_ particles, enabling single‐electron transfer (SET) to activate electrophilic trifluoromethylation reagents such as Umemoto reagents.^[^ ^64^, ^65^, ^66^ ^]^ The resulting trifluoromethyl radicals selectively added to aromatic compounds, including *N*‐heterocyclic and electron‐rich systems.^[^ ^29^ ^]^ Oxidation of the intermediate by polarized BaTiO_3_ and subsequent deprotonation restored the aromatic system. The radical pathway was confirmed by isolating a TEMPO ((2,2,6,6‐Tetramethylpiperidin‐1‐yl)oxyl)‐trapped *N*─CF_3_ intermediate **11** (Figure 3, middle). The reaction was conducted under liquid‐assisted grinding (LAG) conditions, where polar solvents like acetonitrile, DMF, or acetone were essential for achieving satisfactory yields, while nonpolar solvents like hexane proved ineffective. Using tetragonal BaTiO_3_ as the piezoelectric material, 3‐methylindole (**10**) was trifluoromethylated at the C‐2 position in 62% yield within 90 min at 30 Hz. In contrast, cubic BaTiO_3_, with its high symmetry and lower net polarization under mechanical impact, was significantly less effective.^[^ ^67^ ^]^ Furthermore, control experiments under conventional solution‐based conditions in acetone (0.3 M) at room temperature with stirring for 24 h produced no product, confirming that mechanical agitation was essential for particle polarization and efficient radical trifluoromethylation.^[^ ^29^ ^]^ This mechanochemical approach demonstrated broad applicability, successfully trifluoromethylating various *N*‐heterocyclic substrates in yields up to 75%. Notably, the sleep‐wake cycle hormone melatonin was trifluoromethylated at its C‐2 position in 65% yield (product **12**). The method's feasibility for postsynthetic transformations of biologically active peptide substrates was also validated, as a range of tryptophan‐containing peptides were functionalized (products **13** and **14**).^[^ ^68^, ^69^, ^70^, ^71^, ^72^ ^]^ This trifluoromethylation protocol highlights the versatility of mechanochemical approaches for late‐stage functionalization, offering green and efficient solutions for introducing fluorine‐containing motifs into bioactive molecules.

Moving beyond (trifluoro)methylation, the scope of mechanochemical methods extends to other critical transformations in organic synthesis, particularly transition metal‐catalyzed cross‐coupling reactions for C─C bond formation. Among these, the Suzuki–Miyaura reaction has emerged as one of the most extensively studied under mechanochemical conditions, with numerous groups exploring its potential.^[^ ^73^, ^74^, ^75^, ^76^, ^77^, ^78^, ^79^, ^80^, ^81^, ^82^, ^83^, ^84^ ^]^

**Figure 4:**
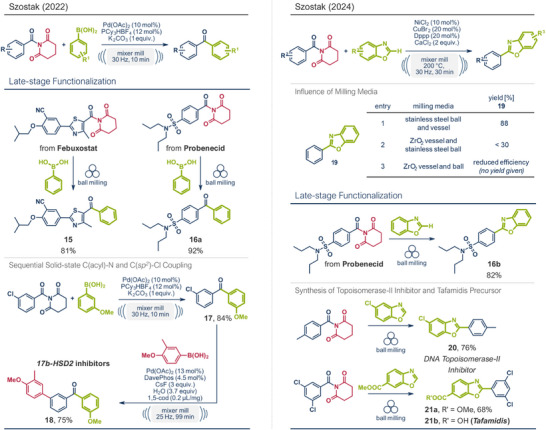
Suzuki–Miyaura cross‐coupling of amides with boronic acid derivatives via selective C─N cleavage (left)^[^ ^44^ ^]^ and C(*sp^2^*)‐C(*sp^2^*) cross‐coupling via triple C─N, C─C, and C─H activation (right) under ball milling conditions by the group of Szostak. Adapted with permission.^[^ ^44^, ^49^ ^]^ © 2021 Wiley‐VCH GmbH and © 2024 American Chemical Society.

Although most protocols utilized aryl halides as coupling partners, the group of Szostak broke new ground by developing a system for the mechanochemical Suzuki–Miyaura cross‐coupling of amides with boronic acid derivatives via selective C─N cleavage (Figure 4, left).^[^ ^44^ ^]^ This solvent‐free protocol is remarkable not only for its high selectivity in σ‐C─N bond activation but also for its short reaction times (<10 min.), air stability, room temperature conditions, and excellent functional group tolerance. Cost‐effective and readily available Pd(OAc)_2_ with PCy_3_HBF_4_ ligand constituted the catalytic system, while K_2_CO_3_ was identified as the optimal base. Control experiments confirmed the necessity of both the palladium species and the ligand for successful coupling. Interestingly, using a stainless‐steel ball significantly outperformed ZrO_2_ milling balls, though the underlying rationale was not discussed. The excellent functional group compatibility of this method enabled high‐yielding late‐stage functionalizations of known pharmaceuticals. For example, the antigout drug febuxostat and the antihyperuricemic agent probenecid were first prefunctionalized by converting their carboxylic acid groups into *N*‐acyl‐glutarimides, which then served as coupling partners. Using the established ball milling protocol, these derivatives were converted into benzoyl drug analogues **15** and **16a** in yields of 81% and 92%, respectively. To further demonstrate the protocol's utility for pharmaceutical applications, Szostak's team successfully synthesized *17β‐HSD2* inhibitors for osteoporosis treatment through a two‐step Suzuki–Miyaura coupling sequence. The first step proceeded via C─N bond activation to generate a benzoyl derivative (84% yield), while the second step via C(*sp^2^*)─Cl bond activation to yield the desired product **18** in 75%.

Recently, Szostak's group expanded the application of *N*‐acyl‐glutarimides in mechanochemical, transition metal‐catalyzed coupling reactions, this time accessing aryl‐heteroaryl products through a unique sequence of triple C─N, C─C, and C─H activation (Figure 4, right).^[^ ^49^ ^]^ Notably, this reaction diverges from their previous protocols producing benzoyl derivatives, as CO was released during the transformation. The catalytic system comprised NiCl_2_ (10 mol%), CuBr_2_ (20 mol%), and 1,3‐bis‐(diphenylphosphino)propane (Dppp, 20 mol%) as a ligand, with CaCl_2_ playing a crucial role. This additive likely served a dual purpose: preventing amide hydrolysis by adsorbing water and aiding C─H activation of the benzoxazole. Alternative bases such as CsF, Cs_2_CO_3_, or K_3_PO_4_ proved inefficient. The solvent‐free nature of this reaction mitigates concerns over explosion hazards posed by flammable solvent–gas mixtures at high temperatures, a feature particularly advantageous given the reaction's requirement for 200 °C. However, such elevated temperatures may limit the protocol's compatibility with heat‐sensitive substrates. As seen in their prior studies, the choice of milling materials significantly impacted the reaction yield of **19**; substituting the stainless‐steel milling ball with one made of ZrO_2_ resulted in a significant yield decrease from 88% to 30%. This effect was attributed to the lower density of ZrO_2_ (5.9 g cm^−3^) compared to stainless steel (7.9 g cm^−3^), which reduces kinetic energy impact. Though the authors excluded catalytic involvement of Fe–Cr steel material, the decreased efficiency observed with both ZrO_2_ milling balls and jars leaves room to speculate about possible secondary influences from stainless steel. Despite these considerations, the protocol demonstrated high efficiency, enabling the coupling of various aryl amides and heterocyclic compounds in just 30 min. The utility of this method was further showcased in the late‐stage functionalization of probenecid (product **16b**), as well as in the synthesis of a DNA topoisomerase‐II inhibitor **20** and the pharmaceutically active compound tafamidis **21b**. Although the atom economy of using *N‐*acyl‐glutarimides may not be optimal, the ability to indirectly couple readily available carboxylic acid derivatives after preactivation (amide formation) greatly expands the pool of viable coupling partners in nickel‐catalyzed C─C bond‐forming reactions.

**Figure 5:**
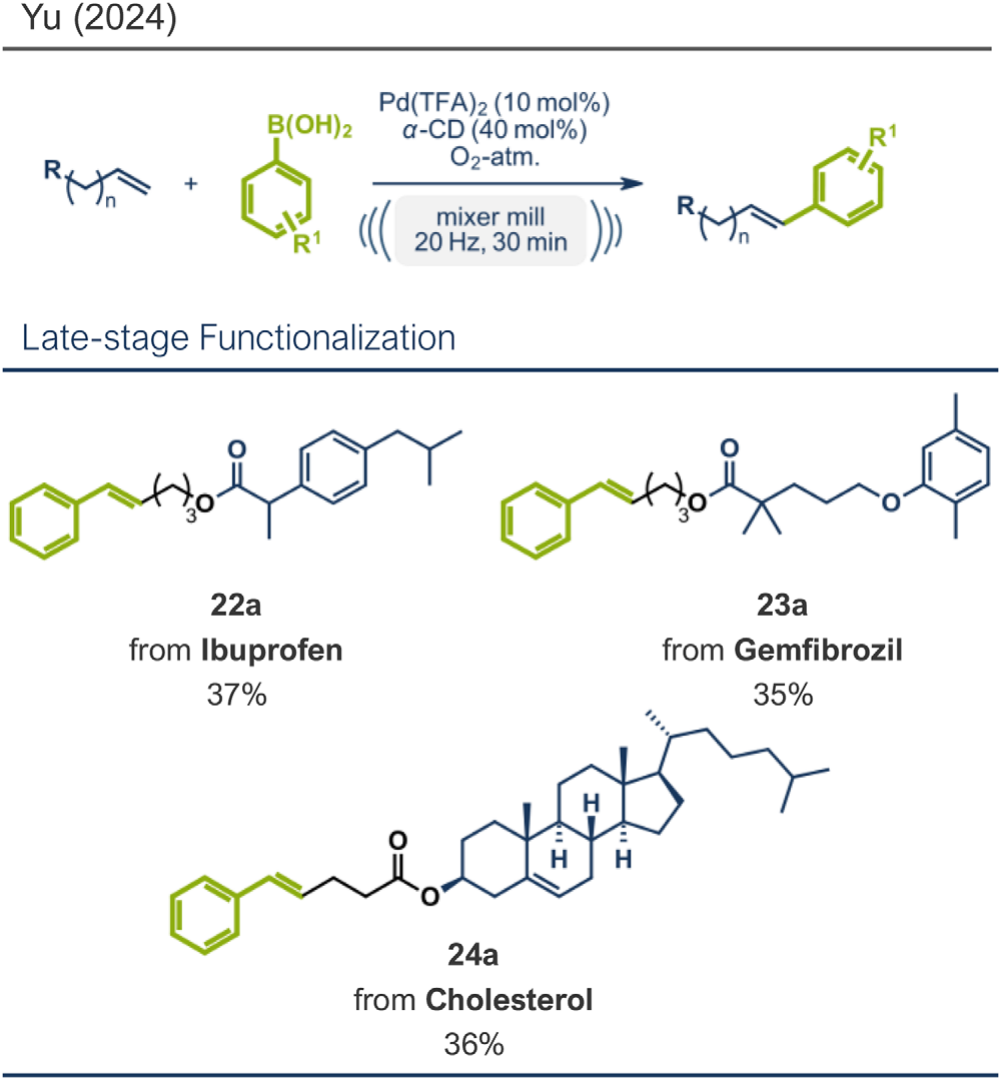
Oxidative Heck coupling under polymer‐assisted grinding conditions in a ball mill by the group of Yu. Adapted with permission.^[^ ^37^ ^]^ © 2024 Royal Society of Chemistry.

Regarding palladium‐catalyzed cross‐coupling reactions, the Mizoroki–Heck reaction has also been extensively studied under mechanochemical conditions.^[^ ^85^, ^86^, ^87^, ^88^, ^89^, ^90^ ^]^ Its closely related variant, the oxidative Heck coupling, has gained attention for enabling regioselectivity control and greener synthesis strategies.^[^ ^37^, ^91^ ^]^ Challenges in these systems include the use of inactive alkenes, which can lead to inseparable mixtures of double‐bond isomers due to reinsertion of metal‐hydride species and subsequent *β‐*hydride elimination.^[^ ^92^, ^93^ ^]^ To address these issues, Yu and coworkers recently developed an elegant approach for mechanochemically driven oxidative Heck coupling, employing polymer‐assisted grinding (POLAG) (Figure 5).^[^ ^37^ ^]^ This strategy leverages polymer additives to stabilize catalysts or influence molecular interactions during mechanochemical reactions.^[^ ^81^, ^87^, ^94^ ^]^ Remarkably, Yu's group discovered that using commercially available cyclodextrins (CDs) as POLAG agents enabled regioselective control in the oxidative coupling of phenylboronic acids with unbiased olefins.^[^ ^37^ ^]^ Cyclodextrins interacted with both substrates and the palladium catalyst, forming a stabilized metal‐CD complex in situ. This interaction not only provided regioselectivity but also aligned with green chemistry principles by utilizing molecular oxygen as the sole oxidizing agent, renewable CDs, and a reusable catalytic system.^[^ ^4^, ^5^, ^18^ ^]^ They used Pd(TFA)_2_ as readily available catalyst together with 0.4 equivalents of *α‐*cyclodextrin under oxygen atmosphere to obtain the desired styrene derivatives in up to 82% yield in a short 30‐min ball milling process at 30 Hz. Although this method did not directly functionalize drug molecules, it demonstrated compatibility with biologically active compounds through premodifications. For instance, bioactive compounds such as ibuprofen, gemfibrozil, and cholesterol were esterified to bear terminal alkenyl moieties, enabling coupling with phenylboronic acid (products **22a**, **23a**, and **24a**). Impressively, the internal double bond of modified cholesterol remained unaltered during the reaction, exemplifying the protocol's regioselectivity and its potential for precise functionalization of sensitive molecules.

**Figure 6:**
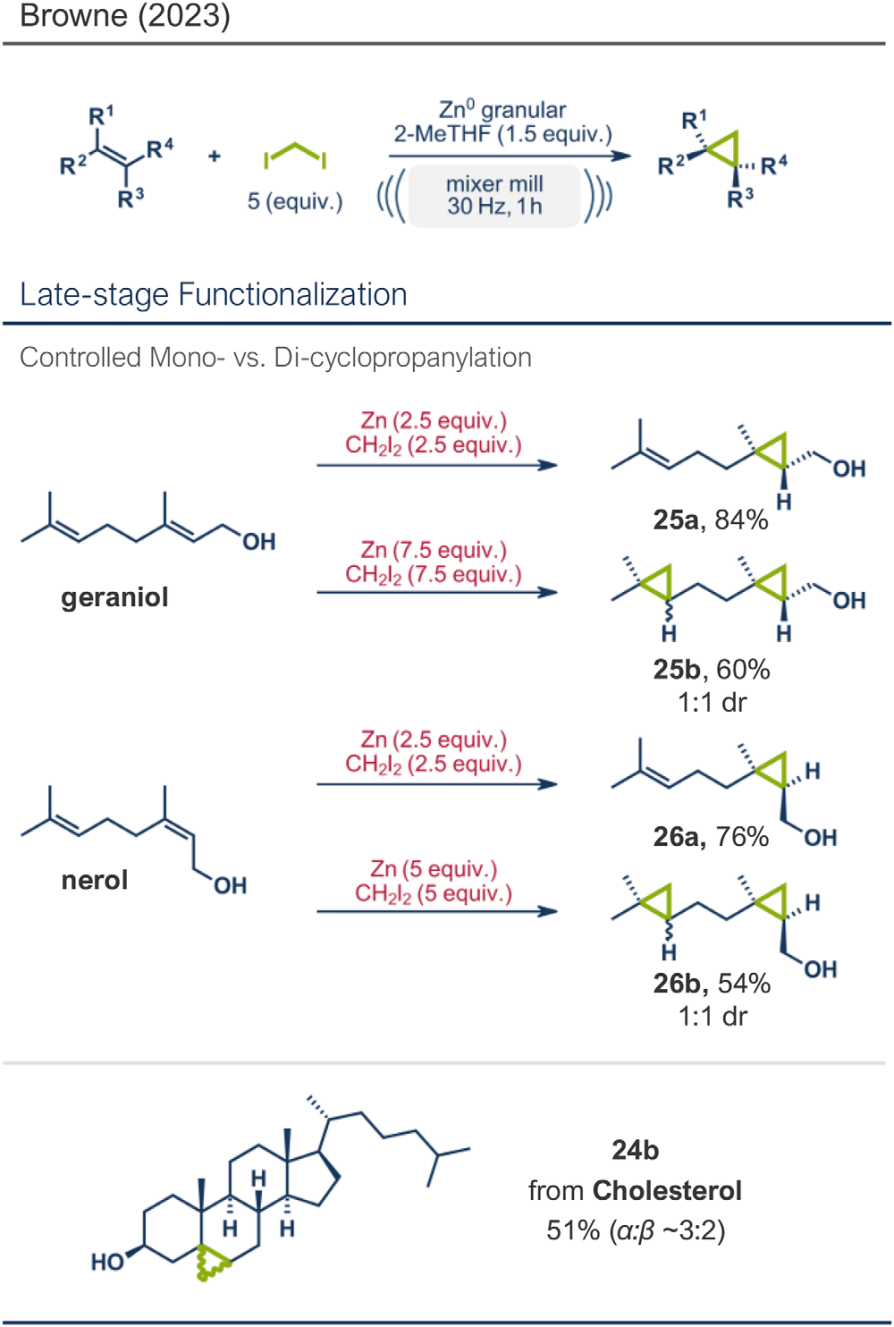
Mechanochemical Simmons–Smith cyclopropanation by the group of Browne. Adapted with permission.^[^ ^36^ ^]^ © 2023 Royal Society of Chemistry.

A different strategy for efficient C─C bond formation involves the reaction of organometallic nucleophiles, generated via metal insertion into carbon─halide bonds, with electrophiles. In traditional solution‐based processes, these nucleophiles are typically prepared in situ under strictly air‐ and moisture‐free conditions due to their high reactivity. Remarkably, however, mechanochemical conditions often allow these reactions to proceed smoothly under ambient atmosphere. This significant improvement underscores the unique advantages of solvent‐free systems, particularly when generating organometallic species under ball milling conditions. In addition to magnesium,^[^ ^95^, ^96^, ^97^ ^]^ calcium,^[^ ^98^ ^]^ or manganese metals,^[^ ^99^ ^]^ zinc has emerged as a versatile reagent for organometallic transformations. The Browne group has explored the application of organozinc species in various mechanochemical transformations, including the Negishi coupling (2018), Reformatsky reaction (2019), and Barbier‐type allylation (2020).^[^ ^100^, ^101^, ^102^ ^]^ Recently, the group reported a zinc mediated Simmons–Smith cyclopropanation under mechanochemical conditions (Figure 6).^[^ ^36^ ^]^ Their findings consistently demonstrate that mechanical impact from ball milling is critical for activating raw zinc. For instance, attempts to perform the reaction under neat conditions or by stirring in 2‐MeTHF without mechanical force resulted in no observable conversion to the cyclopropanated product, despite otherwise identical reaction conditions. Thermal measurements confirmed that the reaction temperature during ball milling did not exceed 31 °C, affirming that the transformation is driven by mechanical energy rather than heat. Using diiodomethane and zinc powder (5 equiv each) under liquid‐assisted grinding (LAG) conditions with 2‐MeTHF, the protocol enabled the efficient conversion of a wide range of olefins into cyclopropane derivatives within 1 h at 30 Hz and room temperature, with yields reaching up to 95%. Notably, geraniol and nerol – naturally occurring monoterpenoids frequently used in perfumery – could be chemoselectively mono‐ or di‐cyclopropanated by adjusting the stoichiometry of zinc. Lower zinc amounts (2.5 equiv) facilitated selective reaction at the allylic alcohol's double bond, leaving the sterically hindered trisubstituted double bond intact (products **25a** and **26a**), while higher zinc quantities (5–7.5 equiv) resulted in dicyclopropanation (products **25b** and **26b**). Cholesterol, a structurally complex substrate, underwent cyclopropanation in 51% yield under elevated temperatures (75 °C) with sand as a grinding auxiliary (product **24b**). This achievement is particularly noteworthy, as a Simmons–Smith reaction of cholesterol has so far been unsuccessful in solution but proved feasible under mechanochemical conditions.

**Figure 7:**
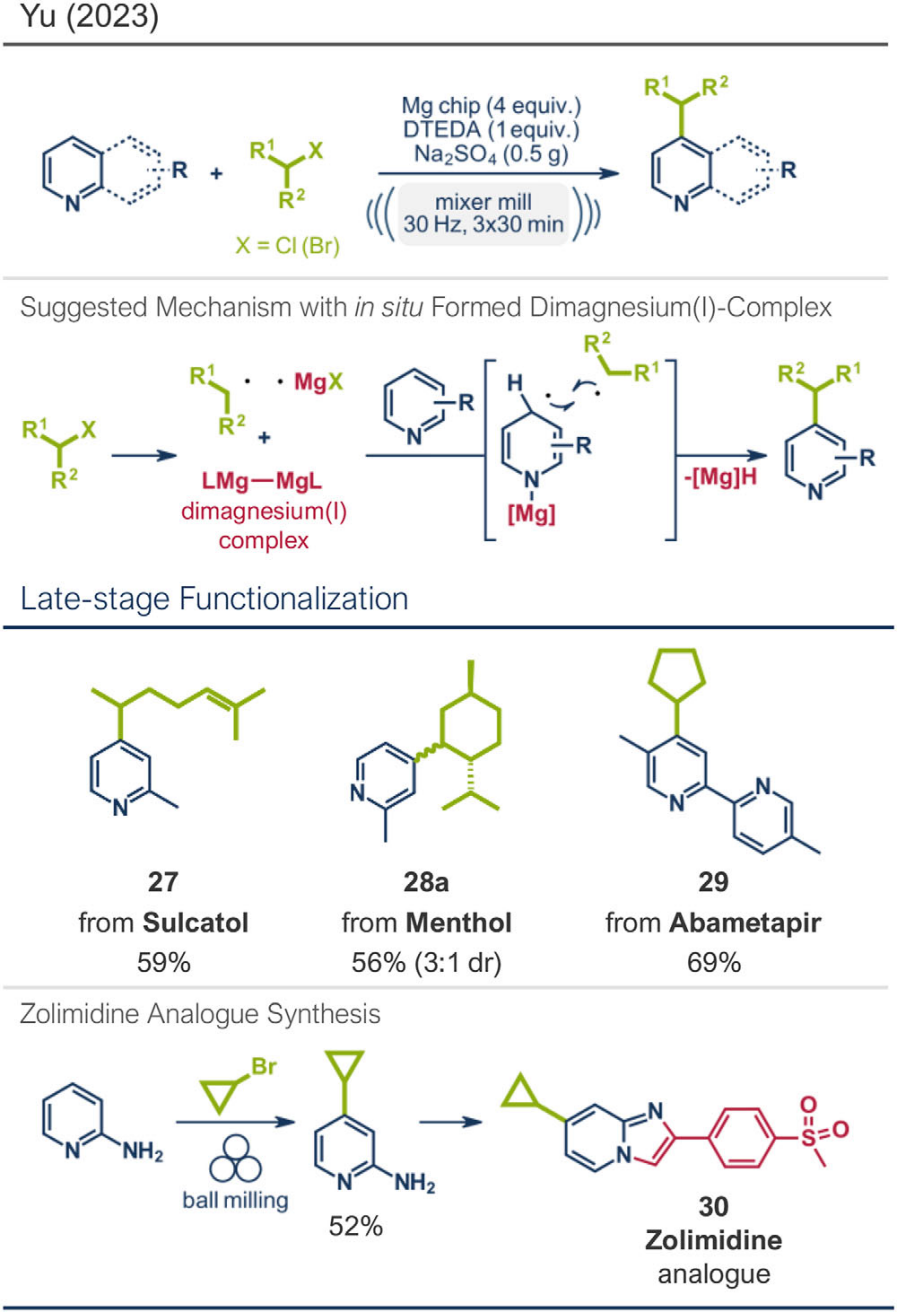
Magnesium‐mediated radical C─H alkylations of *N*‐heterocyclic substrates using ball milling by Yu and coworkers. Adapted with permission.^[^ ^27^ ^]^ © 2023 American Chemical Society.

Expanding the scope of zero‐valent base metal‐mediated transformations, Yu and coworkers investigated magnesium‐mediated radical C─H alkylations of *N*‐heterocyclic substrates (Figure 7).^[^ ^27^ ^]^ Building on their earlier work on mechanochemically induced radical reactions^[^ ^97^, ^103^ ^]^ and inspired by studies from Wakefield^[^ ^104^ ^]^ and Jones,^[^ ^105^ ^]^ the group hypothesized that alkyl radicals generated from alkyl halides via Mg(0), combined with pyridine radicals formed through an in situ low‐valent dimagnesium(I) complex, could unlock a novel route to solvent‐free C‐4 alkylation of pyridine derivatives.^[^ ^27^ ^]^ Using 2‐methylpyridine and chlorocyclohexane as a model system, the reaction proceeded in a mixer mill at 30 Hz over 90 min under ambient atmosphere. The main challenge in designing the reaction was to maintain the stability and reactivity of the in situ formed dimagnesium(I) complex. It was found that bidentate Lewis bases with bulky substituents, particularly *N,N’*‐di‐*tert*‐butyl‐ethane‐1,2‐diamine (DTEDA), played a crucial role in stabilizing the magnesium(I) species, thus preventing its disproportionation. The reaction required an excess of magnesium metal (5 equiv) and Na_2_SO_4_ as a grinding auxiliary to obtain the desired product in 74% yield with exceptional C‐4 regioselectivity. Mechanistic studies provided strong evidence for the involvement of radical intermediates and suggested that the C─H bond cleavage of pyridine was the rate‐determining step (Figure 7, middle). This method enabled the successful coupling of primary, secondary, and tertiary alkyl halides with various pyridine derivatives, leading to the formation of modified biologically active compounds from menthol (product **28a**), sulcatol (pheromone, product **27**), and abametapir (metalloproteinase inhibitor, product **29**). Additionally, the method was applied to the synthesis of zolimidine analogue **30** through C‐4 cyclopropanylation of 2‐aminopyridine followed by a subsequent one‐step transformation.

**Figure 8:**
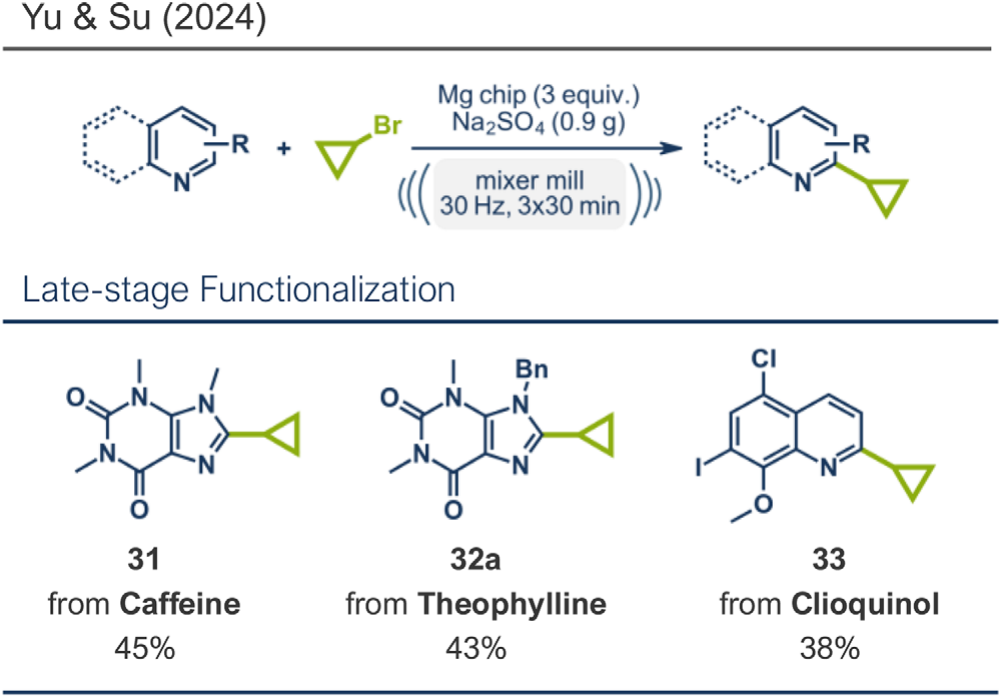
Magnesium‐mediated Minisci C─H cyclopropanylation using ball milling by Yu, Su and co‐workers. Adapted with Permission.^[^ ^34^ ^]^ © 2024 Royal Society of Chemistry.

Very recently Yu, Su, and coworkers developed a mechanochemical, three step synthesis toward the cholesterol‐lowering agent pitavastatin.^[^ ^34^ ^]^ *En route* they investigated a magnesium‐mediated Minisci C‐H cyclopropanylation under similar reaction conditions to their previously reported C‐4 pyridine alkylation (Figure 8). Excess zero‐valent magnesium activated cyclopropylbromide under ball milling conditions, with Na_2_SO_4_ serving as a crucial grinding auxiliary. In this case, the pyridine substrates were already substituted at the C‐4 position, which directed the cyclopropanylation to occur exclusively at the C‐2 position. Similar to the “magic methyl effect” (vide supra), late‐stage cyclopropanylation of bioactive compounds can modify their properties, enhancing metabolic stability, lipophilicity, and specificity.^[^ ^106^ ^]^ To demonstrate the applicability of their method for the late‐stage modification of bioactive compounds, Yu and coworkers successfully applied their solvent‐free, mechanochemical protocol to cyclopropanylate caffeine (CNS stimulant, product **31**), *N‐*benzylated theophylline (phosphodiesterase inhibitor, product **32a**), and *O‐*methylated clioquinol (antiseptic, product **33**).^[^ ^34^ ^]^

**Figure 9:**
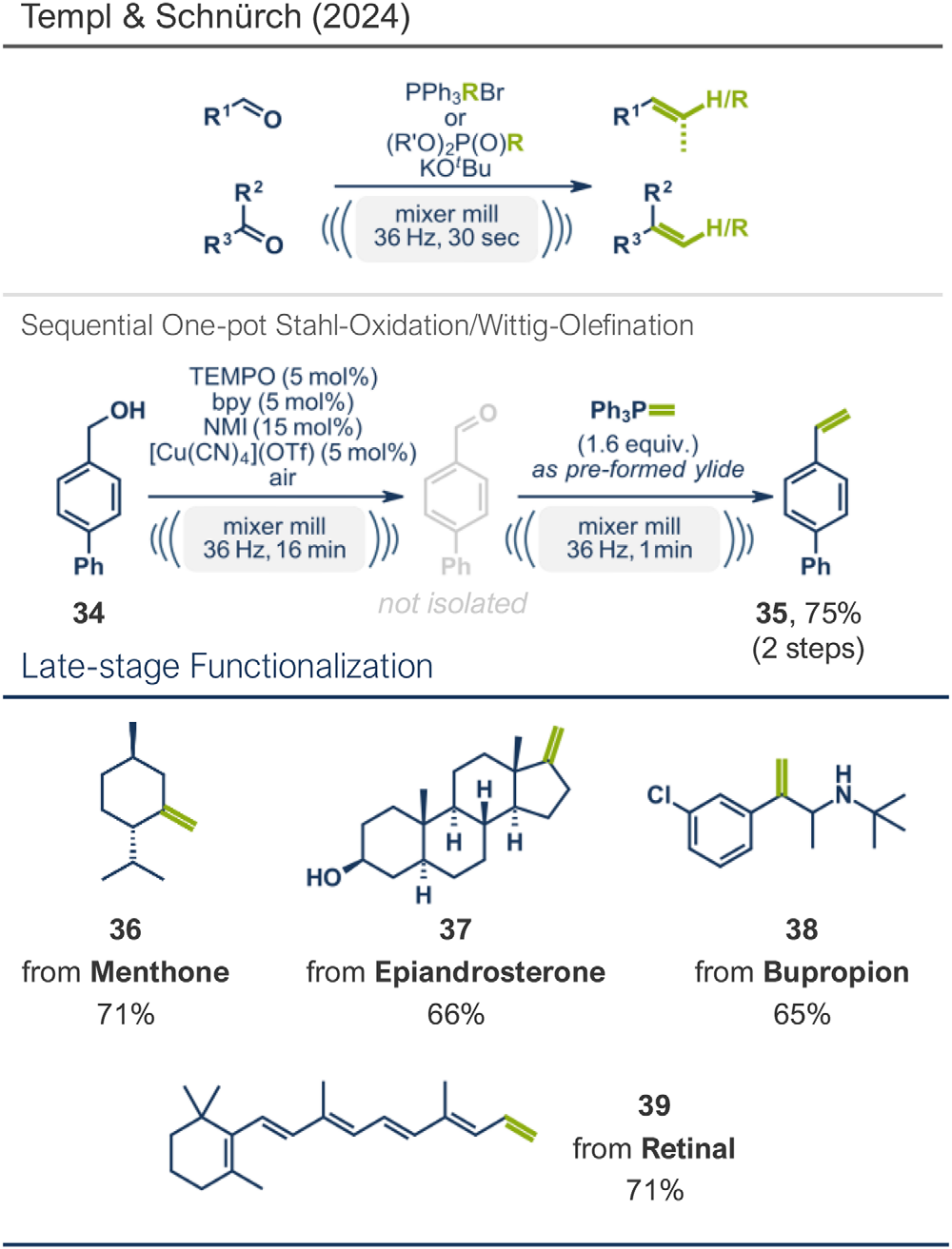
Wittig‐Olefination and sequential one‐pot oxidation/olefination protocol under ball milling by Templ and Schnürch. Adapted with permission.^[^ ^33^ ^]^ © 2024 Wiley‐VCH GmbH.

The Wittig reaction, a key transformation for converting aldehydes and ketones into double bonds, is widely applied in synthetic chemistry, including BASF's industrial‐scale synthesis of Vitamin A and its derivatives.^[^ ^107^, ^108^, ^109^, ^110^, ^111^ ^]^ Conventional solution‐based methods often require tedious ylide preformation at low temperatures, followed by heating to complete the reaction, making the process labour‐intensive and time‐consuming. Templ and Schnürch recently introduced a mechanochemical, solvent‐free version of this classic reaction that simplifies operations and drastically reduces reaction times (Figure 9).^[^ ^33^ ^]^ Their ball milling approach often eliminates the need for ylide preformation, allowing the direct combination of the phosphonium species, KO*^t^* Bu, and the substrate in a milling vessel, with a reaction time of just 30 s at room temperature. For base‐sensitive substrates, preformation of the ylide can still be performed by milling KO*^t^* Bu and the phosphonium species for 1 min prior to adding the carbonyl compound. The method proved highly versatile, converting a wide variety of structurally diverse substrates into their alkenylated derivatives with high overall yields. The protocol was successfully applied to bioactive molecules such as retinal and menthone (products **39** and **36**, as well as marketed pharmaceuticals including epiandrosterone (androgenic, product **37**) and bupropion (antidepressant, product **38**). To enhance the synthetic utility further, the authors developed a sequential one‐pot oxidation/olefination protocol, enabling the direct transformation of alcohols into olefins without intermediate isolation (cf. alcohol **34** to olefin **35**). The initial oxidation step was adapted from a mechanochemical Stahl‐oxidation protocol reported earlier by Porcheddu's group.^[^ ^112^ ^]^

## 3 C─N Bond Forming Reactions

Nitrogen‐containing structural motifs are privileged in pharmaceutical compounds, with small‐molecule drugs containing an average of over two nitrogen atoms per drug, underscoring their critical role in medicinal chemistry.^[^ ^113^, ^114^ ^]^ These functional groups profoundly influence a compounds’ chemical and biological characteristics. Replacing a carbon atom with nitrogen in drug molecules frequently results in improved pharmacological properties, including enhanced potency and better biochemical profiles.^[^ ^115^, ^116^, ^117^ ^]^ Moreover, nitrogen‐containing functionalities enhance solubility, binding affinity, and selectivity by facilitating hydrogen bonding and ionic interactions with biological targets.^[^ ^114^, ^118^ ^]^ These attributes are essential for optimizing the pharmacokinetics and pharmacodynamics of APIs. Late‐stage nitrogen incorporation into drug molecules offers distinct advantages, enabling the fine‐tuning of pharmacological properties and the development of novel therapeutic agents with superior potency.^[^ ^16^, ^119^, ^120^ ^]^ When coupled with mechanochemical methods, this strategy provides a sustainable alternative for chemical processes in pharmaceutical development. Several studies have demonstrated the feasibility of mechanochemical C─N bond‐forming reactions for synthesizing APIs or their intermediates.^[^ ^121^, ^122^, ^123^, ^124^, ^125^, ^126^, ^127^, ^128^, ^129^, ^130^, ^131^, ^132^, ^133^ ^]^ Complementary, the following sections will detail mechanochemical protocols applied to the late‐stage functionalization of existing pharmaceuticals.

**Figure 10:**
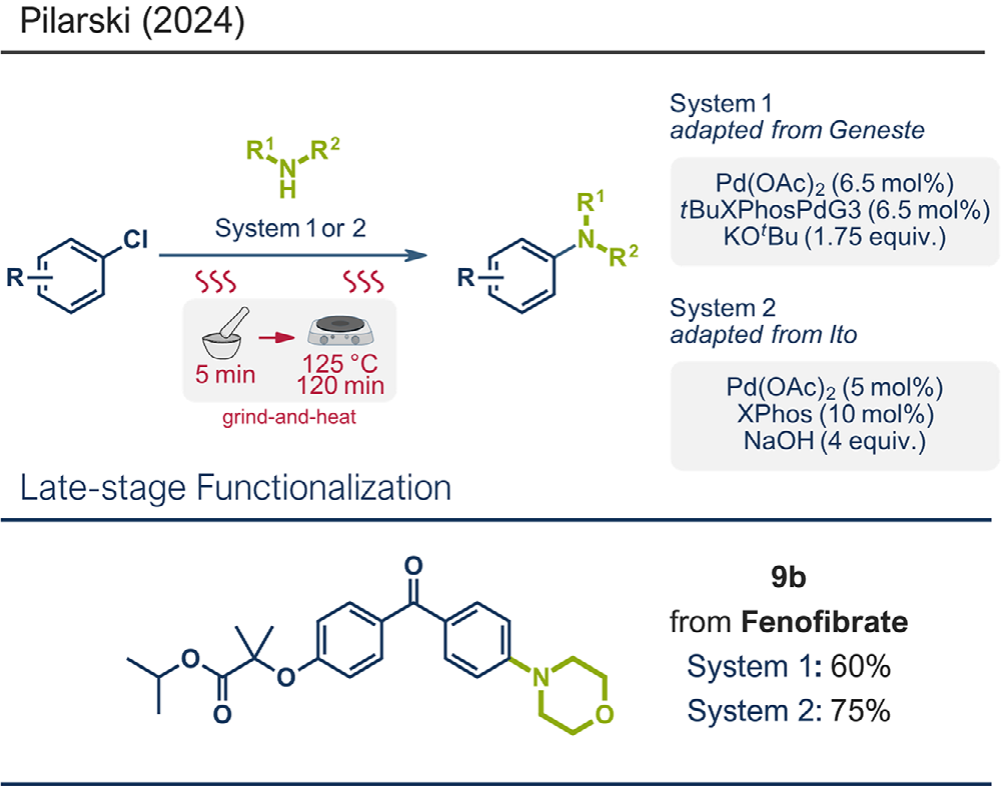
Grind‐and‐heat protocol for Buchwald–Hartwig coupling of C─Cl bonds with secondary amines by the group of Pilarski. Adapted with permission.^[^ ^40^ ^]^ © 2024 Preprint – Cambridge Open Engage (CC BY‐NC‐ND 4.0).

Similar to C─C bond‐forming reactions, several C─N bond forming reactions in mechanochemical synthesis can be enabled by transition metal catalysis. Among these, the Buchwald–Hartwig amination stands out as one of the most prominent C─N cross‐coupling reactions. In recent years, mechanochemical adaptations of the Buchwald–Hartwig coupling have been disclosed by the groups of Ito,^[^ ^134^, ^135^, ^136^ ^]^ Geneste,^[^ ^137^ ^]^ Su,^[^ ^138^ ^]^ and Browne.^[^ ^139^ ^]^ Notably, Su and Browne successfully applied their protocols to the synthesis of drug intermediates, including vilazodone and brexpiprazole in Su's^[^ ^138^ ^]^ and vortioxetine in Browne's work.^[^ ^139^ ^]^ Although these efforts have primarily focused on drug intermediates and not on the modification of existing APIs, they inspired Pilarski's team to explore the potential of adapting existing ball milling protocols for late‐stage drug modification using the previously (see Section 2) described grind‐and‐heat setup (Figure 10).^[^ ^40^ ^]^ Using the conditions established by Geneste^[^ ^137^ ^]^ (System 1) and Ito^[^ ^134^ ^]^ (System 2), Pilarski's team achieved Buchwald–Hartwig coupling of C─Cl bonds with secondary amines, but instead of using a horizontal mixer mill, they performed manual grinding with mortar and pestle (5 min) and subsequently heated the resulting mixture (125 °C for 2 h).^[^ ^40^ ^]^ This approach gave yields comparable to ball milling protocols. Encouraged by these outcomes, they expanded the grind‐and‐heat protocol to late‐stage modifications of pharmaceuticals. For instance, fenofibrate, an antilipemic agent, was coupled with morpholine via its C(*sp^2^*)─Cl bond, giving product **9b** impressive yields of 60% (System 1) and 75% (System 2).

**Figure 11:**
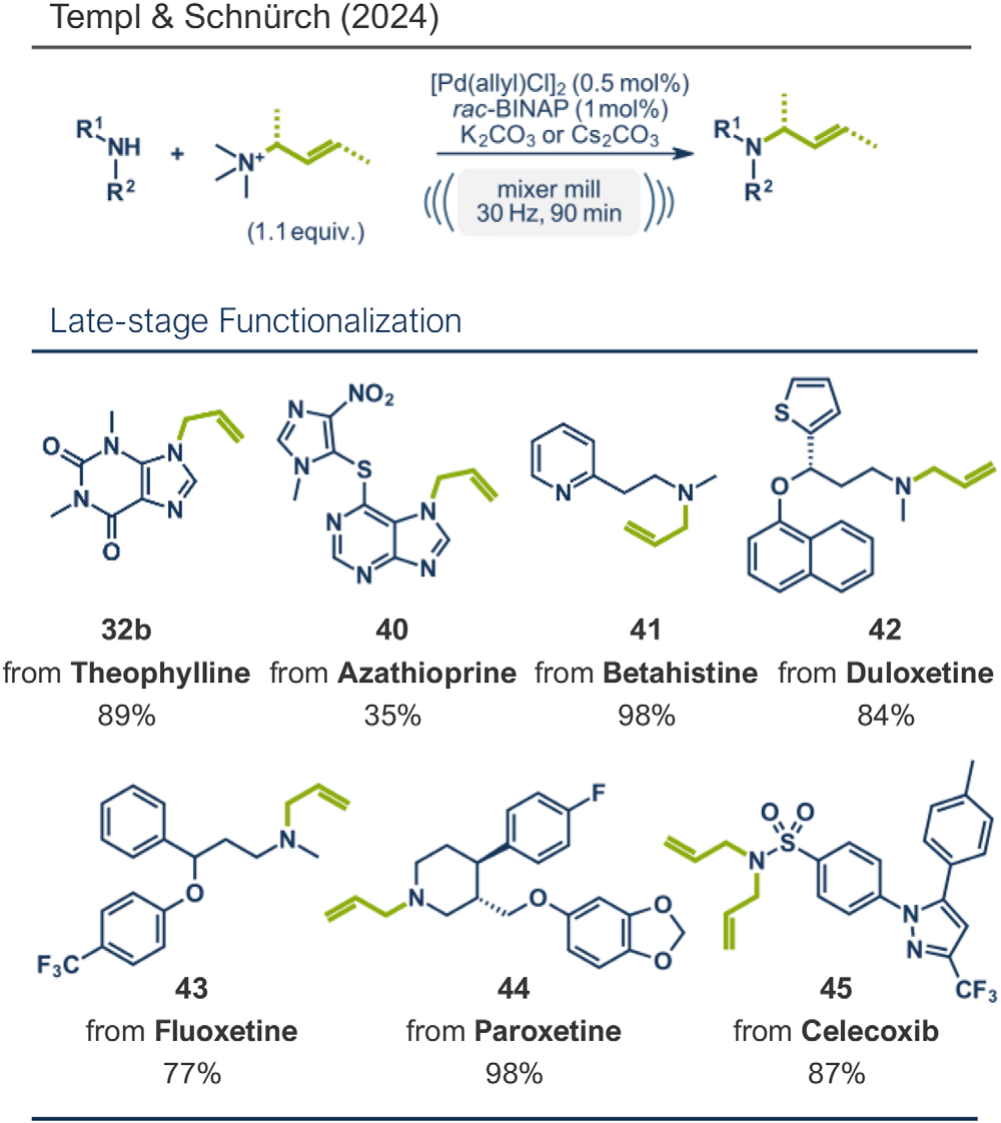
Pd‐catalyzed Tsuji–Trost allylation using quaternary ammonium salts under mechanochemical conditions by Templ and Schnürch. Adapted with permission.^[^ ^30^ ^]^ © 2023 Wiley‐VCH GmbH.

Building upon these advancements, alternative cross‐coupling strategies for mechanochemical C─N bond formation have emerged. One such approach, the palladium‐catalyzed Tsuji–Trost allylation, was recently published by Templ and Schnürch (Figure 11).^[^ ^30^ ^]^ Encouraged by their prior research on quaternary ammonium salts as alternative alkylating agents,^[^ ^140^, ^141^, ^142^, ^143^ ^]^ they utilized solid, easy‐to‐handle allyl ammonium chlorides to synthesize various *O*‐, *N*‐, and *C*‐allylated products.^[^ ^30^ ^]^ This method not only eliminates the need for toxic allyl bromide but also achieves remarkable selectivity for linear products, which would be unattainable with direct use of allyl halides due to competing S_N_2 and S_N_2' reactions. The gaseous and traceless nature of the trimethylamine leaving group simplifies workup and obviating tedious by‐product separation. Employing commercially available [Pd(allyl)Cl]_2_ and *rac‐*BINAP with very low catalyst (0.5 mol%) and ligand (1 mol%) loading together with mild carbonate bases, the reaction proceeded efficiently at room temperature in 90 min at 30 Hz with silica filtration often being sufficient for obtaining pure products in excellent yields. Their method proved particularly effective in the late‐stage allylation of pharmaceuticals, enabling N‐allylation of compounds such as azathioprine (immunosuppressant, product **40**), betahistine (antihistamine, product **41**), the antidepressants fluoxetine (SSRI, product **43**), duloxetine (SSNRI, product **42**), and paroxetine (SSRI, product **44**), theophylline (phosphodiesterase inhibitor, product **32b**), and celecoxib (antiinflammatory, NSAID, product **45**) with yields up to 98%.

**Figure 12:**
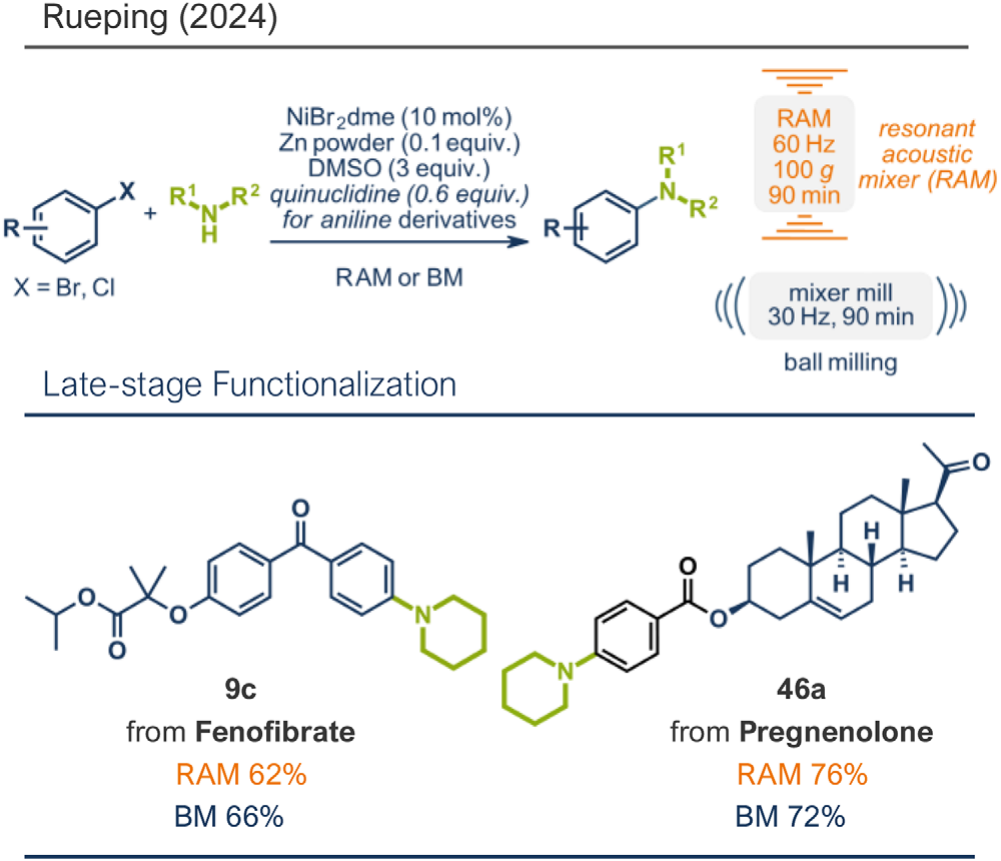
Nickel‐catalyzed cross‐coupling aminations in resonance acoustic mills (RAM) by the group of Rueping. Adapted with permission.^[^ ^45^ ^]^ © 2024 Royal Society of Chemistry.

Recently, the Rueping group systematically investigated nickel‐catalyzed cross‐coupling aminations in resonance acoustic mills (RAM) through high‐throughput screening of reaction conditions (Figure 12).^[^ ^45^ ^]^ The innovative setup involved placing a 96‐well plate into a specially designed holder for RAM devices, significantly accelerating the screening process compared to traditional vibrational mixer mills, where experiments are typically carried out sequentially. The study evaluated the coupling efficiency of two secondary amines, piperidine and aniline, with 4‐bromoacetophenone under various conditions, including different bases, liquid‐assisted solvents, and metal powders, with (1,2‐dimethoxyethane)nickel dibromide (NiBr_2_dme) serving as the catalyst. Gas chromatography was employed to quantitatively analyze the reactions, identifying the optimal conditions: substoichiometric amounts of zinc as reductant and dimethyl sulfoxide (DMSO) as liquid‐assisted solvent in the absence of a base for piperidine and with additional quinuclidine as a base for aryl amines. The general applicability of this protocol was then tested across a variety of structurally diverse substrates bearing different functional groups. To compare the efficacy of mechanical energy input, the authors assessed yields obtained via resonance acoustic mixing (RAM) at 60 Hz and ball milling with a horizontal mixer mill at 30 Hz. The results demonstrated that both methods were effective, with RAM offering slightly better performance for certain substrates. This optimized protocol was subsequently applied to pharmaceutically active compounds. For fenofibrate, piperidine‐coupled product **9c** was obtained in 62% yield using RAM and 66% yield with ball milling. Furthermore, a *p‐*chlorobenzoic acid ester derivative of pregnenolone was successfully coupled with piperidine, achieving yields of 76% and 72% for RAM and ball milling (product **46a**), respectively, showing the potential of these methods for late‐stage modification of drugs and pharmaceutical synthesis.

Metal‐free protocols for mechanochemical C─N bond formations in late‐stage functionalization offer a significant advantage by eliminating residual metal‐traces in modified pharmaceuticals, simplifying purification processes. Over the years, several groups have developed such mechanochemical protocols for amide formation, applying them to the synthesis of APIs like moclobemide (antidepressant),^[^ ^28^, ^122^, ^123^, ^144^ ^]^ lidocaine (local anesthetic),^[^ ^123^ ^]^ and teriflunomide (potential MS medication).^[^ ^129^ ^]^ However, only two groups have explored these protocols for the late‐stage modification of existing bioactive molecules.

**Figure 13:**
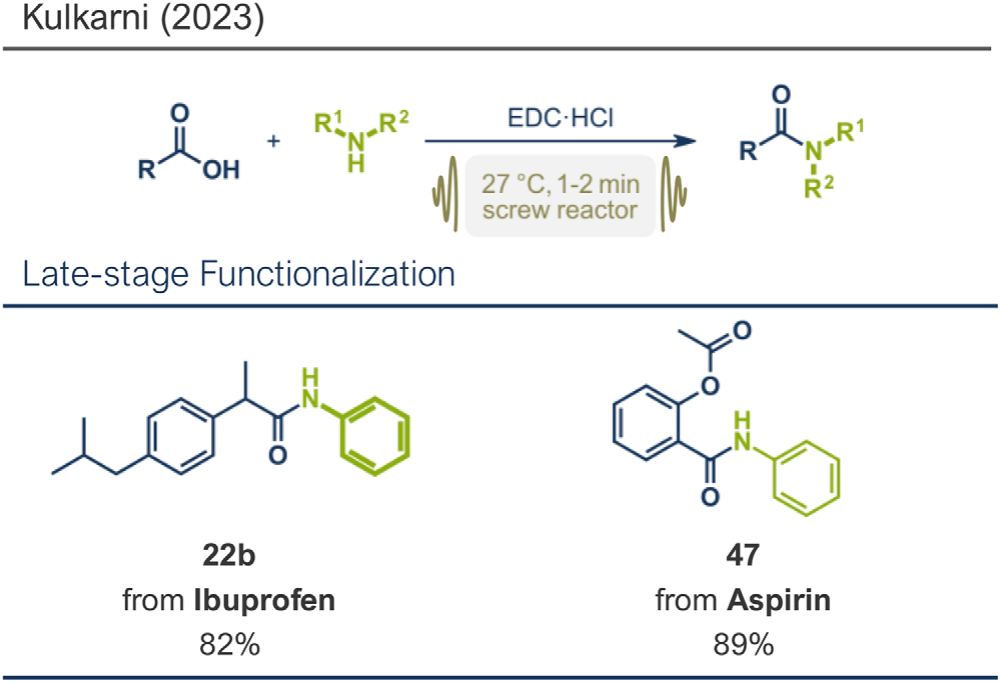
Direct amidation of acids using twin‐screw extrusion for mechanochemical flow synthesis. Adapted with permission.^[^ ^28^ ^]^ © 2023 Royal Society of Chemistry.

In 2023, Atapalkar and Kulkarni reported a solvent‐free protocol for the direct amidation of acids using twin‐screw extrusion for mechanochemical flow synthesis (Figure 13).^[^ ^28^ ^]^ This method utilized 1‐ethyl‐3‐(3‐dimethylaminopropyl)carbodiimide hydrochloride (EDC·HCl) in stoichiometric amounts as the coupling agent, enabling access to structurally diverse amides in yields reaching 95%. The mild room‐temperature conditions, combined with a short residence time of just 1–2 min and a conversion rate of approximately 50 g per h, underscore the method's potential for industrial‐scale pharmaceutical applications. Notably, widely used nonsteroidal anti‐inflammatory drugs (NSAIDs) ibuprofen and aspirin were successfully modified at their carboxylic acid moieties, yielding the respective anilides in 82% (product **22b**) and 89% yield (product **47**). Also, the protocol was not restricted to aryl amines and benzoic acid derivatives, but secondary aliphatic amines, hydrazines, and nonaromatic carboxylic acids could be successfully coupled in high overall yields.

**Figure 14:**
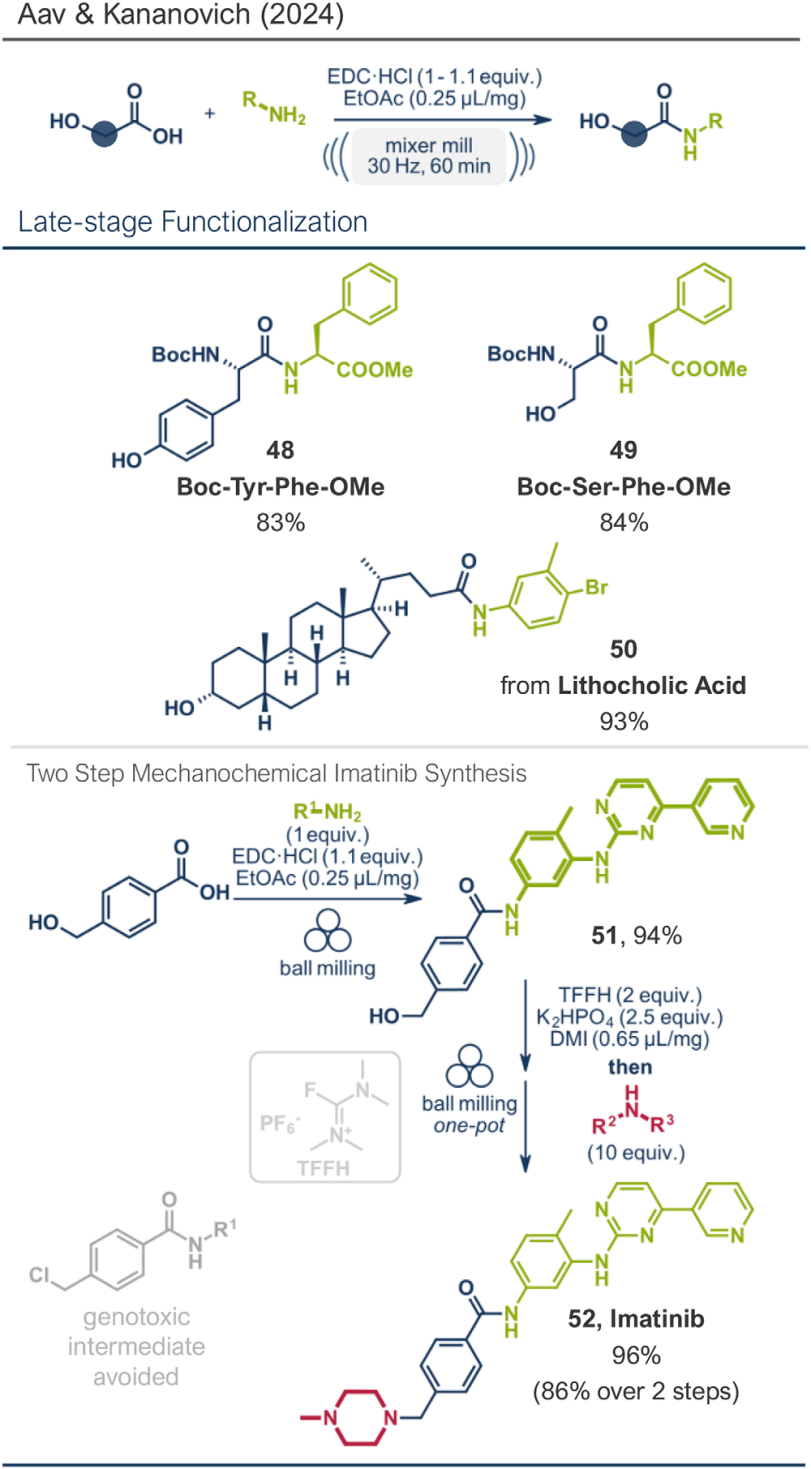
Direct amidation of carboxylic acids by the Aav, Kananovich, and coworkers. Adapted with permission.^[^ ^46^ ^]^ © 2024 Royal Society of Chemistry.

A related protocol using EDC·HCl as the coupling agent was recently reported by Kananovich's group.^[^ ^46^ ^]^ Building on prior research involving alcohol amination with halouronium salts like fluoro‐*N,N,N′,N′*‐tetramethylformamidium hexafluorophosphate (TFFH),^[^ ^145^ ^]^ they developed a complementary chemoselective method for amidation of hydroxycarboxylic acids (Figure 14).^[^ ^46^ ^]^ This approach converted acid functionalities while leaving alcohol groups intact. Utilizing EDC·HCl with ethyl acetate as a liquid‐assisted grinding (LAG) agent (0.25 µL mg^−1^), the method gave access to various aromatic and aliphatic amides in up to 90% yield. Remarkably, Boc‐protected amino acids could be coupled with amino acid ester derivatives and lithocholic acid, a steroid‐type bile acid, was amidated at its acid functionality with the hydroxy group unaltered, achieving an impressive yield of 93%. Kananovich and colleagues combined this acid amidation protocol with their earlier alcohol amination work to develop an efficient synthetic route to the anticancer drug Imatinib (**52**).^[^ ^46^, ^145^ ^]^ Their mechanochemical route featured remarkable chemoselectivity, allowing the use of starting materials containing both hydroxy and acid functionalities. The two‐step process involved EDC·HCl‐mediated acid amidation (product **51**) followed by one‐pot hydroxy activation via TFFH and subsequent amination. This approach gave the target compound **52** in a high overall yield (86% over 2 steps) while avoiding a genotoxic intermediate commonly encountered in solution‐based methods.^[^ ^146^, ^147^, ^148^, ^149^, ^150^, ^151^, ^152^, ^153^ ^]^ This innovative route highlights the advantages of mechanochemical methods for complex pharmaceutical synthesis.

## 4 C─O Bond Forming Reactions

Not only nitrogen, but also oxygen plays a fundamental role in bioactive and pharmaceutical compounds, contributing to their structure and functionality. Incorporated as functional groups like esters, carboxylic acids, alcohols, ethers, or ketones, oxygen imparts unique biological properties to these compounds.^[^ ^154^ ^]^ Oxygen's high electronegativity facilitates the formation of polar covalent bonds, enabling critical interactions such as hydrogen bonding and dipole–dipole interactions within biological systems.^[^ ^16^, ^155^ ^]^ These interactions are often essential for modulating a compound's bioavailability, solubility, or binding affinity to a specific biological target, thus influencing its pharmacokinetics and pharmacodynamics.^[^ ^156^ ^]^ The nature of the oxygen‐containing functional group in drug molecules often determines their therapeutic use, with ether and ester groups, for instance, often functioning as prodrugs, releasing active compounds in a controlled manner through in vivo hydrolysis.^[^ ^157^, ^158^, ^159^, ^160^ ^]^ Hydroxy groups enhance water solubility and can participate in hydrogen bonding with macromolecules like enzymes or receptors,^[^ ^156^, ^161^ ^]^ while ketones and aldehydes can act as recognition motifs in enzymatic binding.^[^ ^162^, ^163^ ^]^ Given the prevalence of oxygen‐containing functional groups and the potential of prodrug approaches, late‐stage modification to incorporate new C─O bonds offers vast opportunities in drug discovery and optimization. In the following chapter, we will discuss innovative mechanochemical strategies for constructing C─O bonds which were applied in the late‐stage modification of bio‐ and pharmacologically active compounds.

Classical solution‐based chemistry has established numerous methods for etherification, including palladium‐catalyzed cross‐coupling reactions,^[^ ^164^, ^165^, ^166^, ^167^ ^]^ Ullmann coupling,^[^ ^168^ ^]^ the Williamson synthesis,^[^ ^169^ ^]^ and the Mitsunobu reaction.^[^ ^170^, ^171^, ^172^, ^173^ ^]^

**Figure 15:**
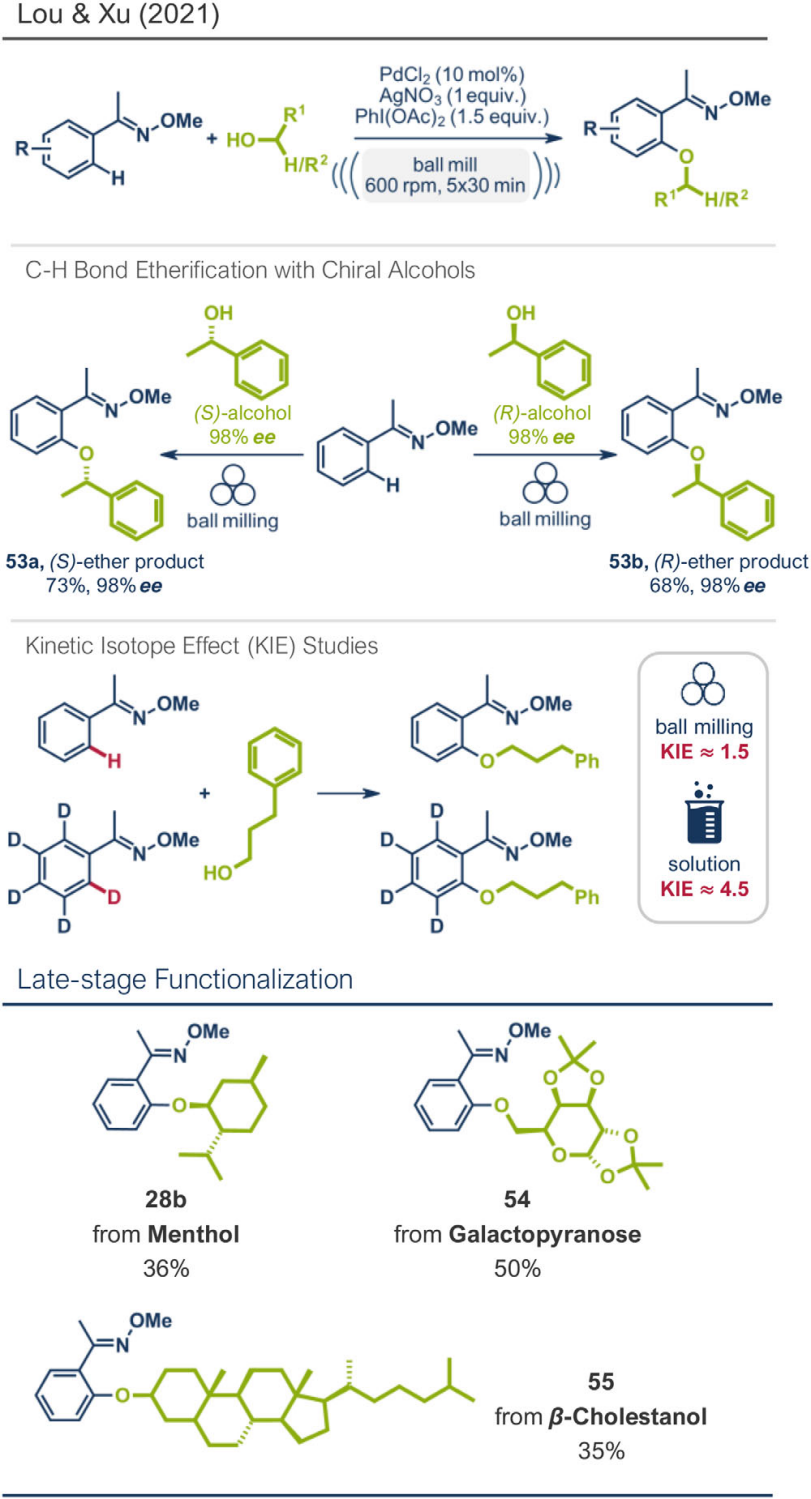
Mechanochemical directing group‐mediated aryl ether formation under palladium catalysis by Lou, Xu, and coworkers. Adapted with permission.^[^ ^35^ ^]^ © 2021 American Chemical Society.

Mechanochemical approaches have emerged as efficient alternatives, with direct activation of C─H bonds under metal catalysis as a particularly atom‐efficient strategy. Lou, Xu, and coworkers applied this strategy to mechanochemically drive aryl ether formation (Figure 15).^[^ ^35^ ^]^ Using oxime ethers as a directing groups, *ortho*‐C(*sp^2^*)─H bonds were activated via palladium catalysis and coupled with primary and secondary alcohols. The addition of AgNO_3_ (0.5 equiv) significantly improved yields, most probably by enhancing palladium's electrophilicity via ionic interactions, while PhI(OAc)_2_ served as stoichiometric oxidant. Unlike solution‐based protocols requiring excess alcohol (often applied as a (co‐)solvent), this approach used near‐stoichiometric amounts (1.5–3 equiv), proving advantageous for costly or complex alcohols. Successful coupling of *β*‐cholestanol, menthol, and galactopyranose with oxime ethers demonstrated the method's applicability for late‐stage etherification (products **55**, **28b**, and **54**). The authors did not explicitly comment on the enantioselectivity of the reaction when employing (+)‐menthol, however, the use of enantiomerically pure *(S)*‐ and *(R)*‐1‐phenylethanol resulted in the formation of the corresponding ethers **53a** and **53b** with high enantiopurity (98% ***ee***), indicating that the method likely enables the general accessibility of enantiomerically pure products. Furthermore, the authors demonstrated that the oxime directing group could be easily converted into the corresponding ketone by treatment with a conc. HCl:DCM mixture at 70 °C, enhancing the method's utility for drug modifications. Kinetic isotope effect (KIE) studies revealed a significant difference in the rate‐determining step when comparing the solvent‐free, mechanochemically driven etherification protocol to a thermally induced aryl ether formation in solution (using dichloroethane as solvent at 70 °C).^[^ ^35^, ^174^ ^]^ For the ball milling approach, a KIE of approximately 1.5 was identified, while the solution‐based reaction exhibited a much higher KIE of around 4.5 (Figure 15, middle). These findings suggest that under solvent‐free conditions, mechanistic changes may occur, with the C─H bond cleavage – the rate‐determining step – being accelerated compared to the conventional solution‐based systems.

**Figure 16:**
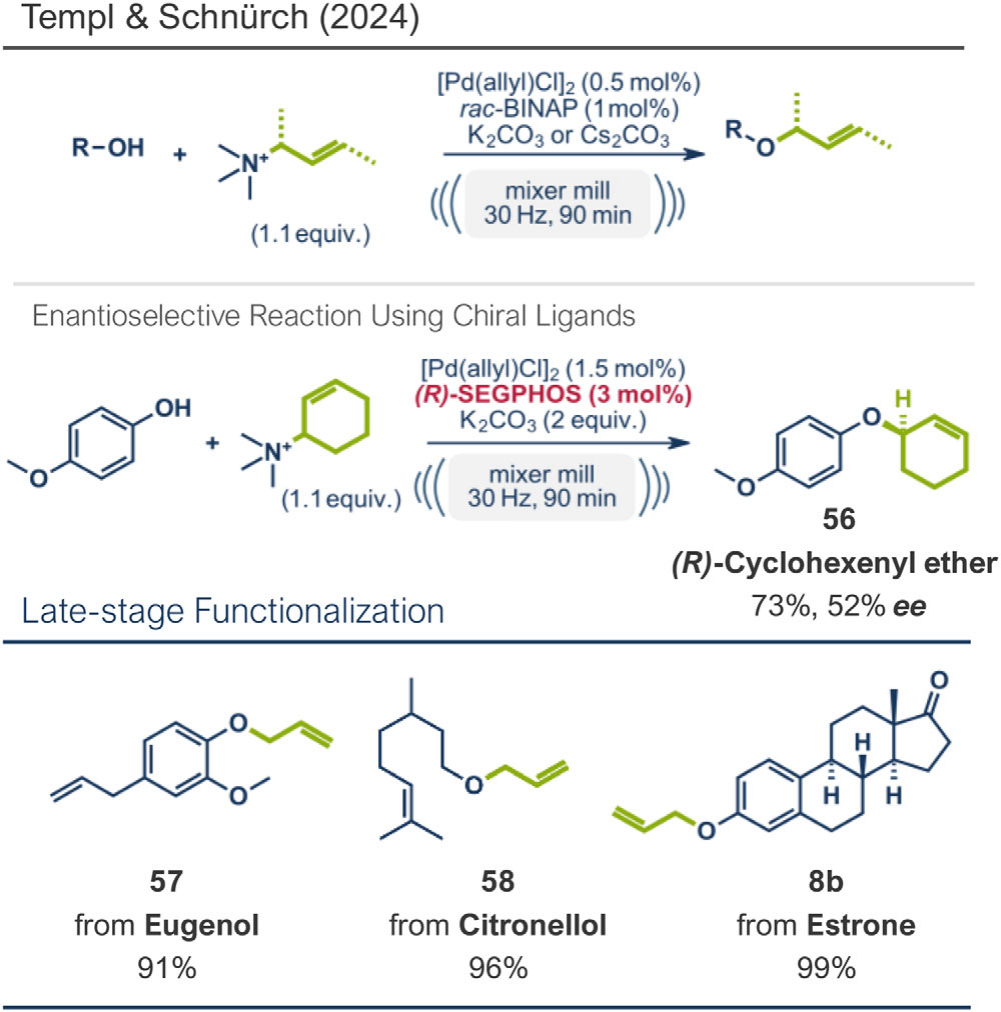
Mechanochemical Tsuji–Trost allylation for allyl ether formation via palladium‐catalysis by Templ and Schnürch. Adapted with permission.^[^ ^30^ ^]^ © 2023 Wiley‐VCH GmbH.

Another palladium‐catalyzed protocol, previously discussed for C─N bond formation, that is also applicable for late‐stage etherification of hydroxy‐containing bioactive compounds, is the mechanochemical Tsuji–Trost allylation using solid quaternary allyl ammonium salts, developed by Templ and Schnürch (Figure 16).^[^ ^30^ ^]^ In this approach, the [Pd(allyl)Cl]_2_ catalyst and *rac‐*BINAP, along with mild bases such as K_2_CO_3_ or Cs_2_CO_3_, enabled the allylation of natural and bioactive compounds like eugenol (product **57**), citronellol (product **58**), and the hormone estrone (product **8b**), obtaining up to quantitative yields under completely solvent‐free conditions. The authors demonstrated that for secondary allyl‐containing ammonium salts, asymmetric induction could be facilitated by using chiral ligands. However, this approach achieved only a moderate *ee* of 52% for cyclohexenyl ether product **56**, providing a starting point for future efforts to enhance enantioselectivity.

**Figure 17:**
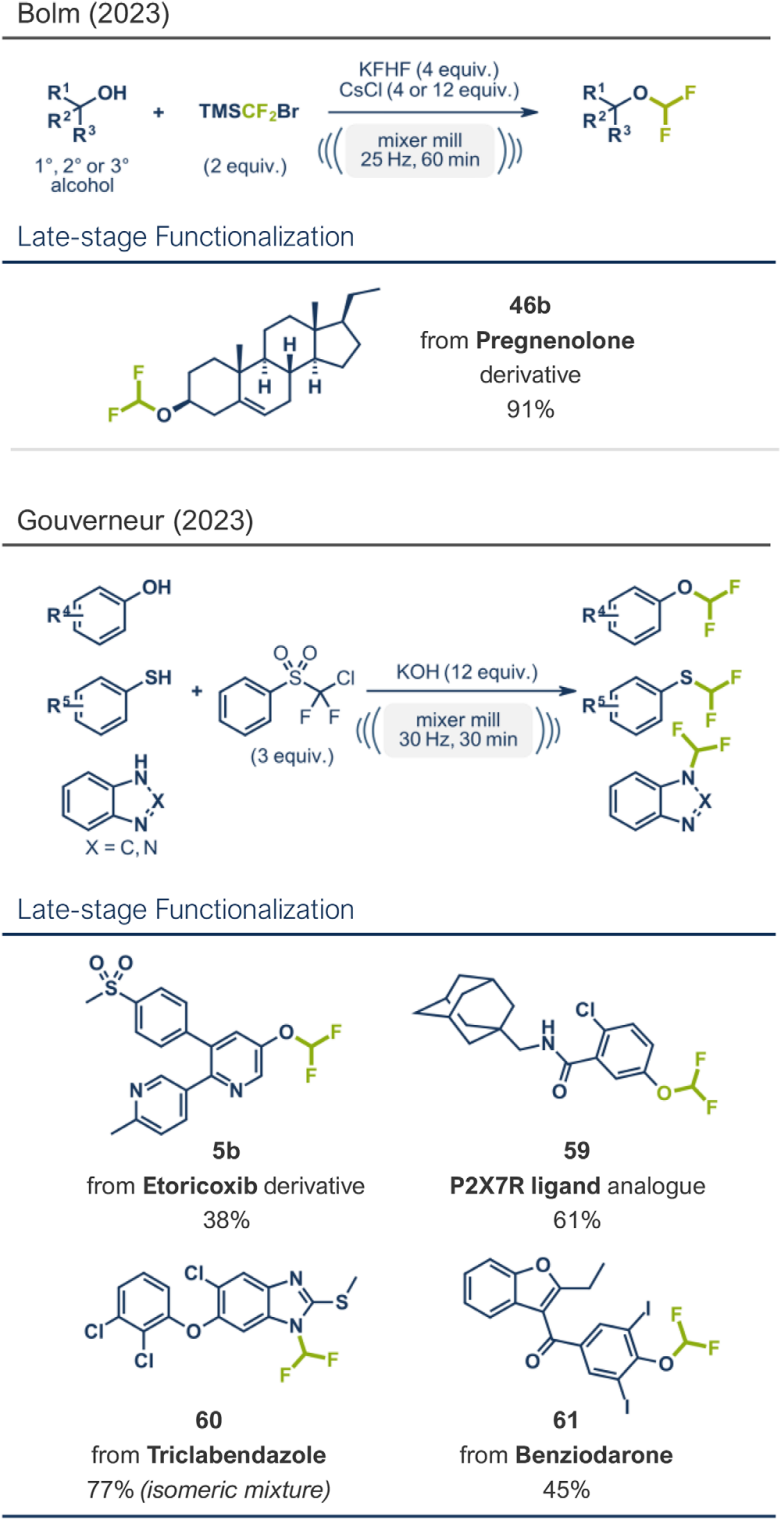
Mechanochemical difluoromethylation protocols by the groups of Bolm^[^ ^48^ ^]^ (top) and Gouverneur^[^ ^31^ ^]^ (bottom). Adapted with permission.^[^ ^31^, ^48^ ^]^ © 2023 Chinese Chemical Society and © 2023 Wiley‐VCH GmbH.

As mentioned previously, the incorporation of fluorine‐containing motifs is a privileged transformation in drug design and modification. The difluoromethoxy group, in particular, has gained significant attention in pharmaceutical research in recent years. This is not only because the difluoromethoxy group can enhance a drug's metabolic stability and lipophilicity, compared to less lipophilic ─OCH_3_, but also because the proton of ─OC**H**F_2_ can still serve as a hydrogen bond donor, a feature that is absent in trifluoromethoxy substituents.^[^ ^175^, ^176^ ^]^ As a result, the development of environmentally friendly protocols for the difluoromethylation of alcohols and phenol derivatives has become an area of great interest. In 2023, both Bolm's group^[^ ^48^ ^]^ and Gouverneur's group^[^ ^31^ ^]^ rather simultaneously reported mechanochemical protocols for achieving difluoromethyl etherifications under solvent‐free conditions. The first protocol, developed by Bolm and his team, utilized TMSCF_2_Br as the difluoromethyl donor along with an excess of KFHF (4 equiv) and CsCl (4 or 12 equiv) as activators (Figure 17, top).^[^ ^48^ ^]^ This method was successfully applied not only to primary but also to secondary and tertiary aliphatic alcohols, converting them to their respective difluoromethyl ethers in up to quantitative yields at 25 Hz, with a reaction time of 1 h. The practicality of the method for late‐stage modification of bioactive compounds was demonstrated by modifying a pregnenolone derivative, yielding the desired product **46b** in 91% yield. However, the difluoromethylation of phenols, imidazoles, and thiols remained challenging. This limitation was overcome by an orthogonal protocol developed by Gouverneur's group (Figure 17, bottom).^[^ ^31^ ^]^ Instead of TMSCF_2_Br, they employed solid and non ozone‐depleting chlorodifluorophenyl sulfone as a difluorocarbene (DFC) precursor. By using a 3‐fold excess of the difluoromethylating agent, along with the hydroxy base KOH, they successfully difluoromethylated a range of phenol and thiol derivatives, as well as *N*‐heterocycles in moderate to good yields. Notably, difluoromethyl ester derivatives of benziodarone (vasodilator, product **61**), etoricoxib (COX‐2 inhibitor, product **5b**), triclabendazole (parasite treatment, product **60**), and a P2X7R ligand analogue (product **59**) were synthesized, further demonstrating the method's potential for late‐stage fluorine installation in pharmaceuticals.

**Figure 18:**
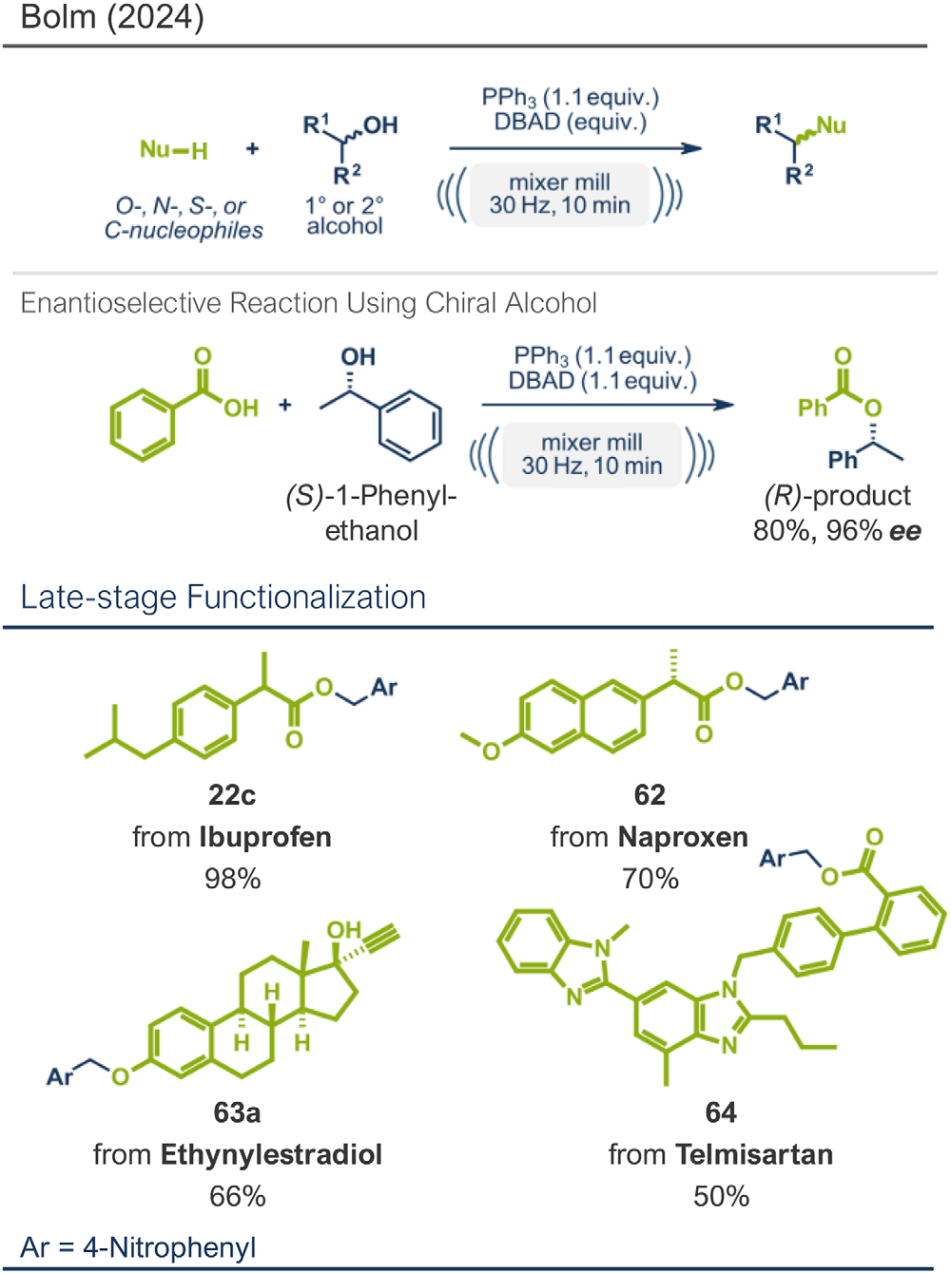
Mechanochemical Mitsunobu reaction by the group of Bolm. Adapted with permission.^[^ ^41^ ^]^ © 2024 Wiley‐VCH GmbH.

Another strategy for accessing C─O bonds is the Mitsunobu reaction. In this reaction, a hydroxy group is displaced by a suitable nucleophile (such as an alcohol, carboxylic acid, etc.) through the initial activation of the hydroxy group with a phosphorus(III) and an azo reagent (e.g., PPh_3_ and diethyl azodicarboxylate (DEAD) system).^[^ ^170^, ^171^, ^172^, ^173^ ^]^ An adaption of the classical Mitsunobu reaction in solution to a solvent‐free mechanochemical approach was recently published by Bolm's group (Figure 18).^[^ ^41^ ^]^ Their system utilized solid, easy‐to‐handle di‐*iso*‐propyl azodicarboxylate (DIAD) and PPh_3_. This method boasts a remarkably short reaction time of only 10 min at 30 Hz using a single 10 mm stainless steel ball in a mixer mill, all under solvent‐free conditions. Both primary and secondary aromatic and aliphatic alcohols were successfully displaced by *O*‐, *N*‐, *S*‐, and *C*‐nucleophiles in yields up to 98%. In general, the Mitsunobu reaction's potential lies in its efficient inversion at the stereogenic center of enantioenriched alcohols. This selectivity was also achieved in Bolm's mechanochemical approach (Figure 18, middle). Using enantioenriched *(S)*‐1‐phenylethanol, they obtained the stereoinverted *(R)*‐product with 96% *ee*. Finally, the versatility and practicality of this method were further demonstrated by the successful late‐stage functionalization of four APIs: naproxen (NSAID, product **62**), telmisartan (angiotensin II receptor antagonist, product **64**), ethinylestradiol (hormone, birth control, product **63a**), and ibuprofen (NSAID, product **22c**).

## 5 C─X Bond Forming Reactions

Halogen substituents are crucial in drug design due to their impact on biological activity. Fluorine, with its high electronegativity, small size, and exceptionally high bond strength to carbon atoms, enhances lipophilicity and metabolic stability, making fluorinated compounds often resistant to enzymatic degradation and desirable in drug development.^[^ ^60^, ^61^, ^62^ ^]^ Iodine, on the other hand, offers unique reactivity due to its polarizability, making it an excellent handle for further chemical transformations. In pharmaceutically active compounds, iodine is also strategically employed to optimize binding interactions or enable radiolabelling for diagnostic applications.^[^ ^177^, ^178^ ^]^ Late‐stage halogenation is particularly valuable for fine‐tuning the properties of drug candidates or exploring structure–activity relationships (SAR) efficiently.^[^ ^179^ ^]^ Mechanochemical methods for C─X bond formation provide a sustainable, solvent‐free way to introduce halogens. This section reviews two such strategies employed in the late‐stage functionalization of bioactive molecules.

**Figure 19:**
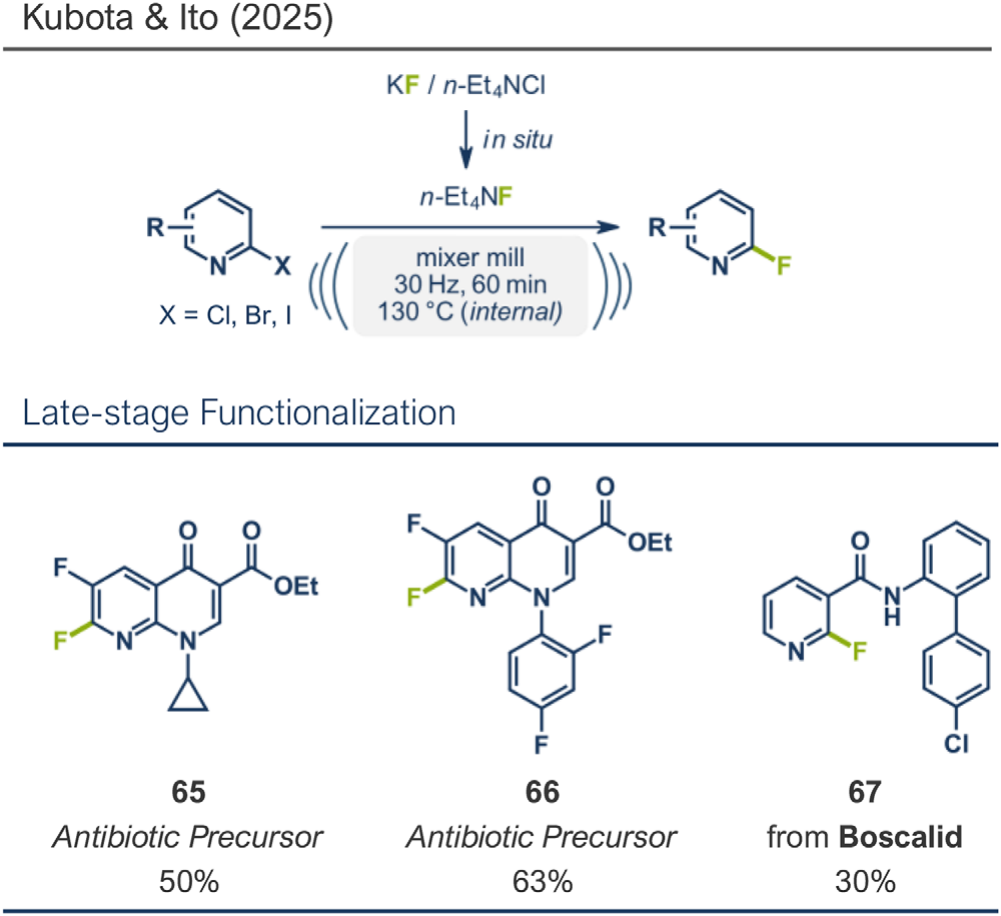
Aromatic nucleophilic fluorination using in situ generated tetramethylammonium fluoride by Kubota, Ito and coworkers. Adapted with permission.^[^ ^32^ ^]^ © Royal Society of Chemistry.

Kubota and Ito recently reported a thermally accelerated aromatic nucleophilic fluorination method in the solid state using quaternary ammonium salts and potassium fluoride (Figure 19).^[^ ^32^ ^]^ By generating the solid‐state fluorinating agent Et_4_NF in situ from KF and Et_4_NCl, they avoided the direct use of moisture‐sensitive tetraalkylammonium fluorides. This ball milling approach is robust and cost‐effective, described as the *“most inexpensive S_N_Ar fluorination yet”*. The ammonium species were key to efficient fluorination, as KF alone was ineffective. However, the reaction required a relatively high internal temperature of 130 °C, limiting its use for heat‐sensitive compounds. Nonetheless, in their model reaction using a 2‐chloroquinoline derivative, fluorination on the former C(*sp^2^*)─Cl bond was achieved in 60 min at 30 Hz quantitatively. A variety of substituted 2‐chloroquinoline derivatives and related structures were well‐tolerated, yielding the respective fluorinated products in high overall yields. In addition to synthesizing two antibiotic precursors (products **65** and **66**), Ito's group successfully applied their protocol for the late‐stage fluorination of boscalid (product **67**), a marketed fungicide. Remarkably, this protocol was not limited to chlorine‐containing substrates and was also feasible with bromo, iodo, and nitro leaving groups, respectively.

**Figure 20:**
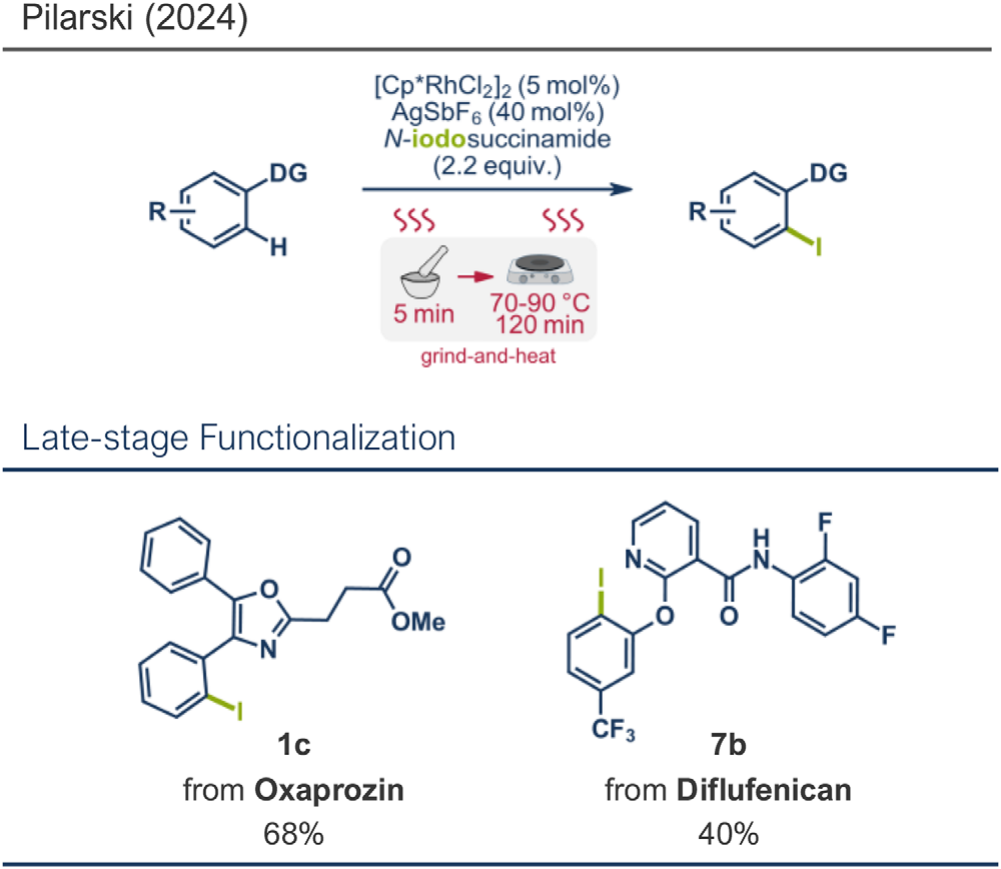
Grind‐and‐heat protocol for ortho‐directed oxidative C─H iodination under ruthenium catalysis by the group of Pilarski. Adapted with permission.^[^ ^40^ ^]^ © 2024 Preprint – Cambridge Open Engage (CC BY‐NC‐ND 4.0).

In analogy to the work of Hernandez and Bolm on *ortho*‐directed oxidative C─H iodination under ruthenium catalysis,^[^ ^180^ ^]^ Pilarski's team investigated the herein reported solid‐state conditions in a grind‐and‐heat setup (Figure 20).^[^ ^40^ ^]^ Without the use of a ball milling reactor, the reagents were manually ground for 5 min with mortar and pestle and subsequently heated to 90 °C for 120 min, yielding 98% of the product, with a yield comparable to Bolm's ball milling protocol. Pilarski's team again focused on late‐stage functionalization of bioactive compounds through the grind‐and‐heat approach using *N*‐iodosuccinamide as the halogenating agent. They successfully iodinated oxaprozin (NSAID, product **1c**) in 68% yield with the oxazoline moiety directing the C─H activation, as well as diflufenican (herbicide, product **7b**) in 40% yield, using the pyridine‐derived substituent as a directing group.

## 6 Miscellaneous Reactions

This section highlights various mechanochemical reactions used for late‐stage modification of pharmaceutically active compounds that have not been covered so far, including bond formation between heteroatoms, multiple bond formations, and reductions.

**Figure 21:**
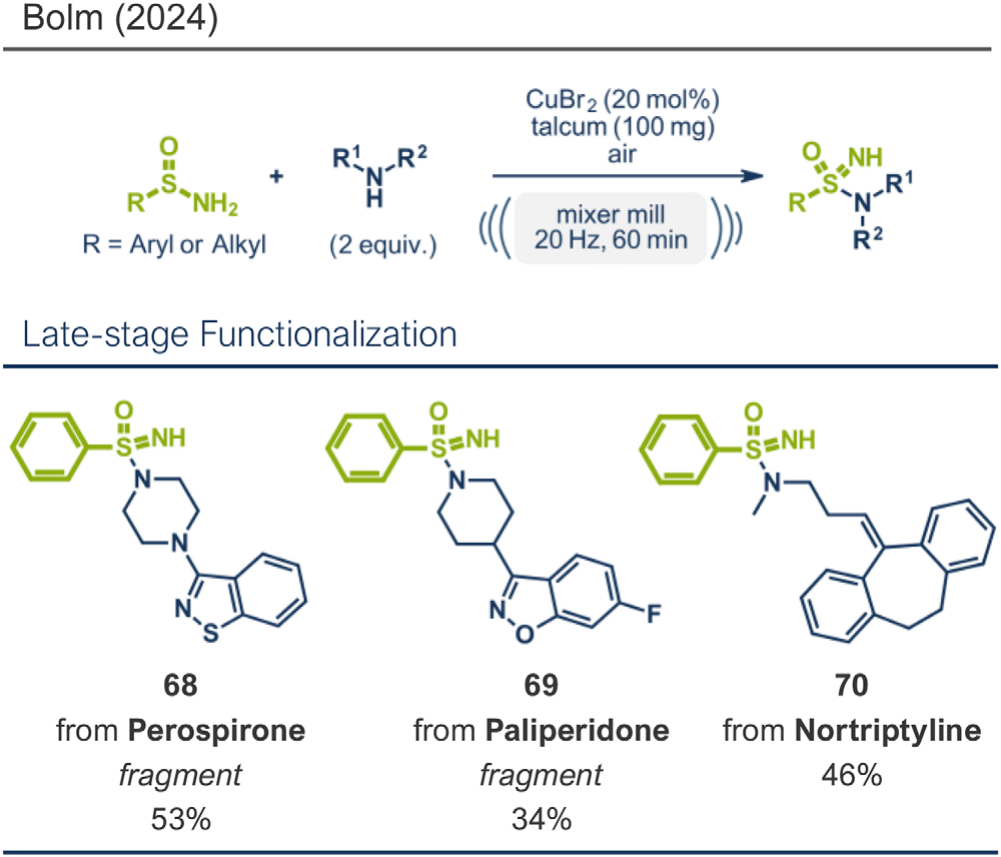
Oxidative amination of primary sulfinamides under copper(II) catalysis in a ball mill by the group of Bolm. Adapted with permission.^[^ ^47^ ^]^ © American Chemical Society.

Bolm and coworkers recently reported a mechanochemical protocol for the oxidative amination of primary sulfinamides under copper(II) catalysis, accessing NH‐sulfonimidamides (Figure 21).^[^ ^47^ ^]^ This solvent‐free method, adapted from a previous solution‐based process,^[^ ^181^ ^]^ used CuBr_2_ as the catalyst and air as the oxidant. Under ball milling conditions (20 Hz, 60 min), yields were comparable to those obtained in a solution‐based process.^[^ ^47^ ^]^ In the absence of the catalyst, the reaction did not proceed, and under an oxygen‐free atmosphere, the conversion remained limited to the molar amount of the catalyst, yielding only around 20% with a single turnover. The method efficiently coupled aromatic and aliphatic primary sulfinamides with primary amines in high average yields. Although sulfinimidamides are not yet marketed drugs, early pharmacological investigations and patents suggest their potential.^[^ ^47^, ^182^, ^183^, ^184^, ^185^ ^]^ A reliable method for the efficient late‐stage functionalization of existing small molecule drugs or their fragments to access novel drug‐like sulfinimidamides is highly desirable. To address this need and demonstrate the potential of their method, Bolm's group successfully coupled benzenesulfinamide with amine‐containing fragments from the APIs perospirone (product **68**) and paliperidone (product **69**). They also performed a late‐stage functionalization of nortriptyline (product **70**), a marketed antidepressant.^[^ ^47^ ^]^

**Figure 22:**
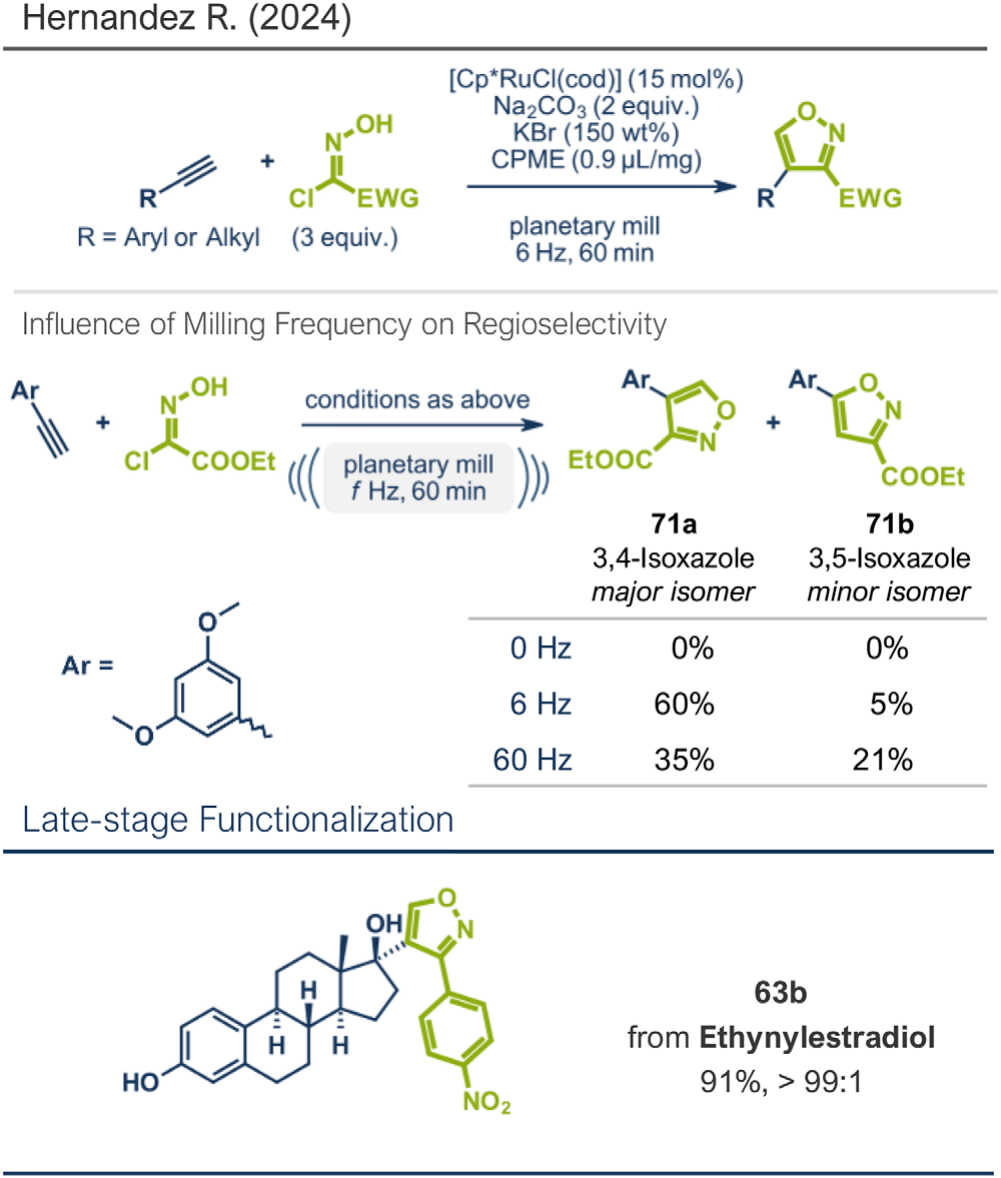
1,3 dipolar cycloadditions in a planetary ball mill for isoxazole synthesis by Hernandez and coworkers. Adapted with permission.^[^ ^42^ ^]^ © 2024 Wiley‐VCH GmbH.

Hernandez et al. reported a mechanochemical protocol for 1,3‐dipolar cycloadditions in a planetary ball mill, forming new C─C and C═N bonds under ruthenium catalysis (Figure 22).^[^ ^42^ ^]^ Hydroxyimidoyl chlorides and alkyls were cyclized, yielding 3,5‐ and 3,4‐substituted isoxazoles, respectively. Detailed studies on the reaction mechanism and parameter screening highlighted the critical role of liquid‐assisted grinding (LAG) agents, which presumably coordinate and stabilize in situ‐generated ruthenium nanoparticles. The method offers excellent regioselectivity control, orthogonal to that observed in copper‐catalyzed systems. Isoxazoles, found in numerous pharmaceutically active compounds,^[^ ^186^, ^187^, ^188^, ^189^ ^]^ also serve as versatile handles for further chemical modifications.^[^ ^190^, ^191^, ^192^, ^193^ ^]^ Interestingly, decreasing the milling frequency to 6 Hz significantly enhanced regioselectivity, favoring 3,4‐isoxazoles (cf. **71a**) over 3,5‐isoxazoles (cf. **71b**).^[^ ^42^ ^]^ This improvement is probably attributed to the catalytic pathway leading to 3,4‐isoxazoles requiring less energy than the concerted pathway yielding 3,5‐isoxazolines. A wide range of isoxazoles from terminal and internal alkynes was synthesized, including an ethinylestradiol derivative (birth control, product **63b**), which was obtained with complete regioselectivity and a 91% yield.^[^ ^42^ ^]^

**Figure 23:**
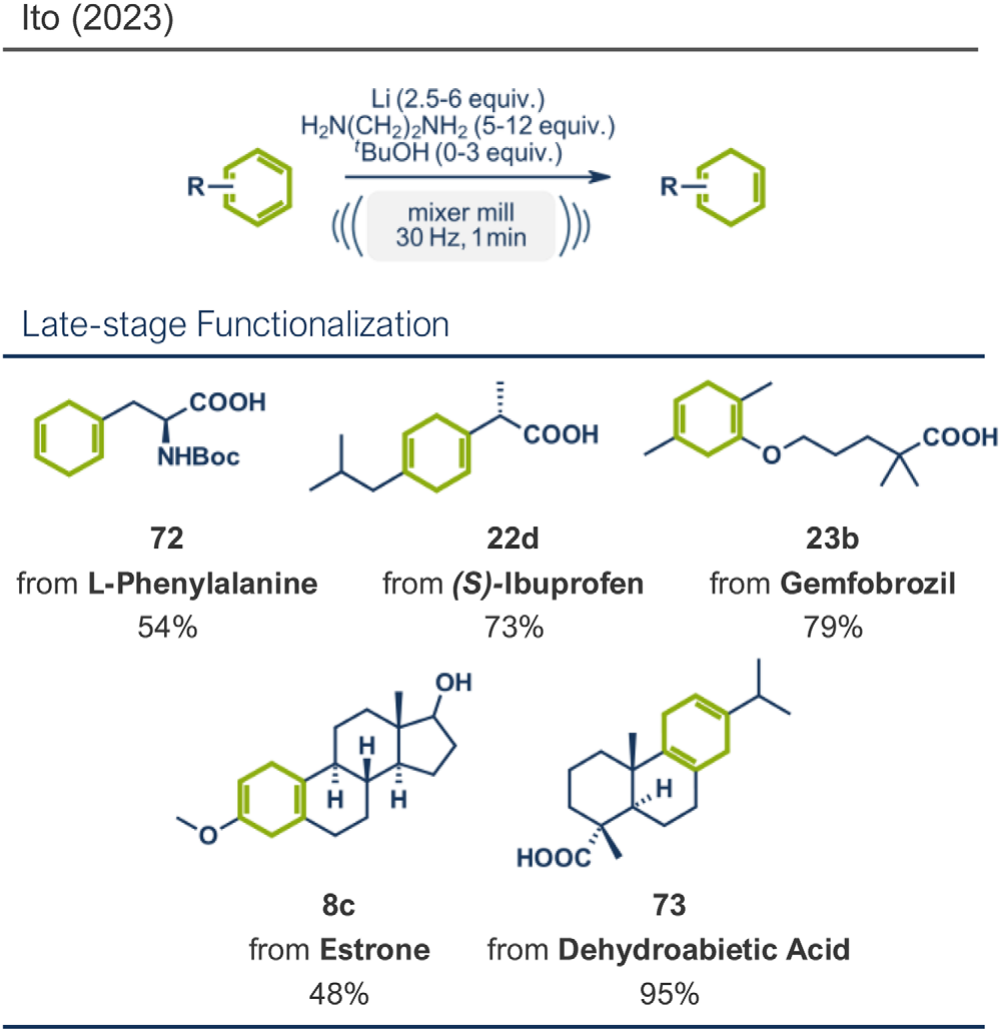
Mechanochemical Birch reduction using lithium metal by the group of Gao. Adapted with permission.^[^ ^38^ ^]^ © 2023 Wiley‐VCH GmbH.

In 2023, Ito's group disclosed a pioneering protocol in the field of mechanochemically driven reduction reactions (Figure 23).^[^ ^38^ ^]^ They developed a novel lithium‐based Birch reduction protocol that is air‐tolerant, NH_3_‐free, and scalable to gram quantities. Unlike conventional Birch reductions, which require low temperatures and strictly inert conditions, this innovative method operates under solvent‐free conditions in air at ambient temperatures using a ball milling reactor. Ethylenediamine (6 equiv) was added to act as a ligand for the lithium metal, which was introduced as a wire with a passivated surface (a thin black oxide film). This passivation prevented the reaction from occurring without mechanical impact. However, during the milling process, the metal surface was activated, enabling it to serve as an effective reductant for arenes. Remarkably, benzoic acid was reduced to its 1,4‐cyclohexadiene derivative in 96% yield after only 1 min of milling. For electron‐rich substrates, a mixture of THF and *t‐*BuOH was added, with the alcohol acting as a proton source to protonate the radical anion intermediate formed during the first single electron transfer (SET) step. The method demonstrated broad applicability, successfully reducing a wide range of arenes with up to quantitative yields. To proof the versatility and utility of their protocol, Ito's team focused on reducing aromatic systems in bioactive compounds and pharmaceuticals. Natural products like l‐phenylalanine (product **72**) and dehydroabietic acid (product **73**), as well as drugs such as *(S)‐*Ibuprofen (NSAID, product **22d**), gemfibrozil (treatment for abnormal blood lipid levels, product **23b**), and estrone (hormone, product **8c**), were dearomatized in high yields while maintaining enantiomeric purity, underscoring the method's applicability in synthetic and pharmaceutical chemistry.

## 7 Beyond Solvent Waste Reduction: The Broader Potential of Mechanochemistry

This review not only aims to comprehensively summarize mechanochemical methods that have proven effective for the late‐stage modification of APIs—demonstrating their compatibility with highly complex molecular scaffolds and their potential applicability in API synthesis itself—but also to highlight unique advantages of mechanochemical protocols over traditional approaches. These distinctive features, particularly relevant for pharmaceutical synthesis and late‐stage API modifications in drug discovery, will be substantiated with examples drawn from the protocols discussed in the main body of the review.

When evaluating environmental metrics such as the environmental factor (E‐factor), the pharmaceutical industry consistently ranks among the least sustainable sectors, largely due to excessive waste generation.^[^ ^194^ ^]^ A major contributor to this issue is the vast amount of solvent waste produced annually—not only during synthesis but also in product purification and reactor cleaning. Mechanochemical protocols, particularly solvent‐free or liquid‐assisted grinding (LAG)‐based approaches, hold significant potential for reducing solvent consumption at the synthesis stage, thereby minimizing overall waste. Although solvent waste reduction is often cited as the primary advantage of mechanochemistry over conventional solution‐based methods, these protocols offer a range of additional unique benefits that make them particularly valuable for drug discovery and pharmaceutical synthesis.

### 7.1 Enhanced Robustness, Simplified Operations, and Increased Rates

In traditional solution‐based synthesis, many organic reactions—especially those involving transition metal catalysis or highly reactive intermediates—require stringent air and moisture exclusion to prevent catalyst or reagent degradation. This in turn means to be dependent on dry and degassed solvents in classical syntheses. On a lab scale the increased costs for these specific solvent requirements might not be immediately apparent. However, in bulk processes, the expense of large quantities of dry and degassed solvents becomes a significant cost factor. Furthermore, sustaining an inert atmosphere is often a second drawback hereby and could be challenging on an increased scale. Several mechanochemical protocols have demonstrated that the absence of bulk solvents significantly enhances reaction robustness, particularly for transformations that are highly air‐ and moisture‐sensitive in solution. This robustness simplifies reaction setup, making mechanochemical methods operationally straightforward and less time‐consuming compared to their solution‐based counterparts.

**Figure 24:**
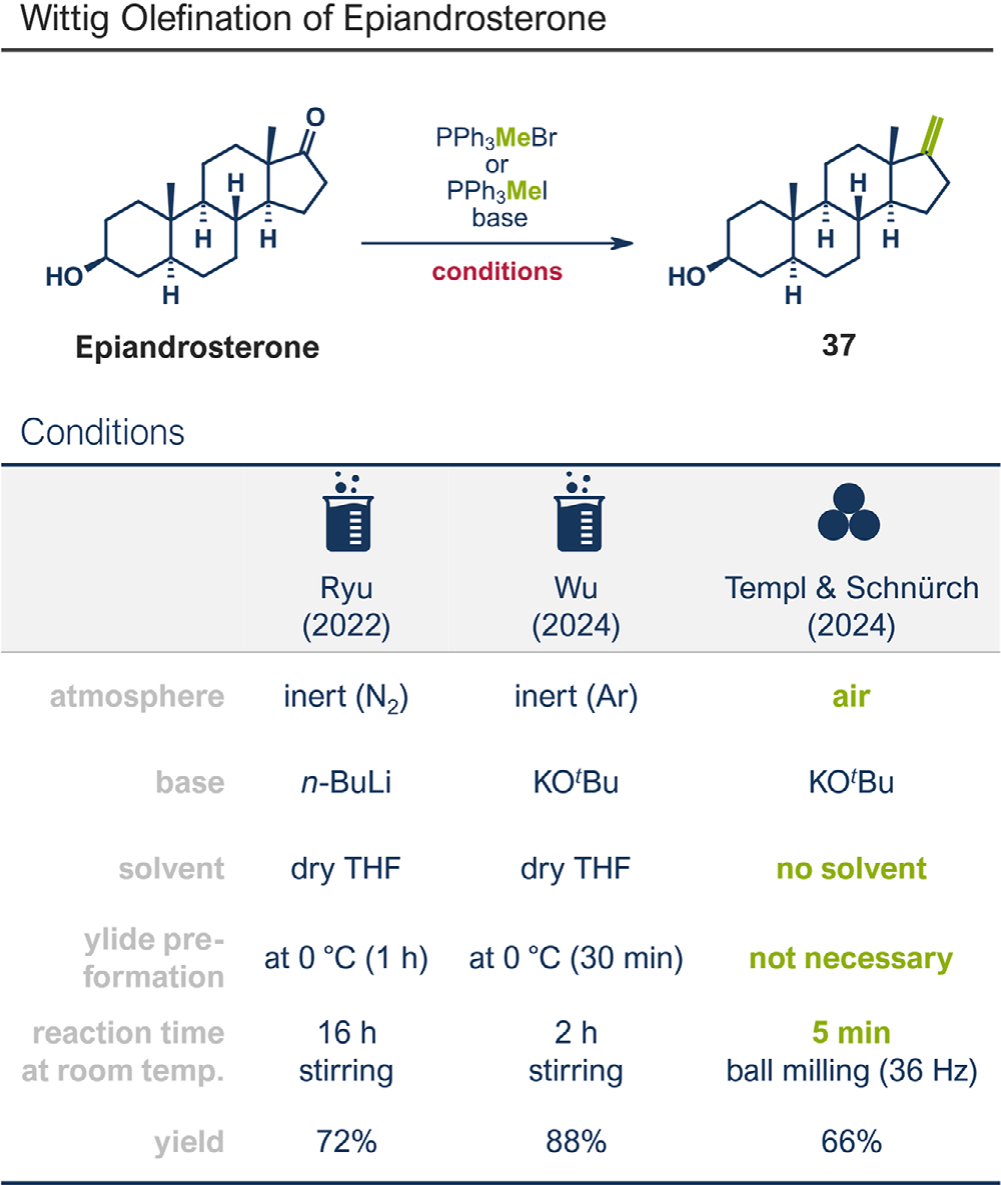
Comparison of solution‐based^[^ ^195^, ^196^ ^]^ versus mechanochemical^[^ ^33^ ^]^ reaction conditions for the Wittig olefination of Epiandrosterone.

**Figure 25:**
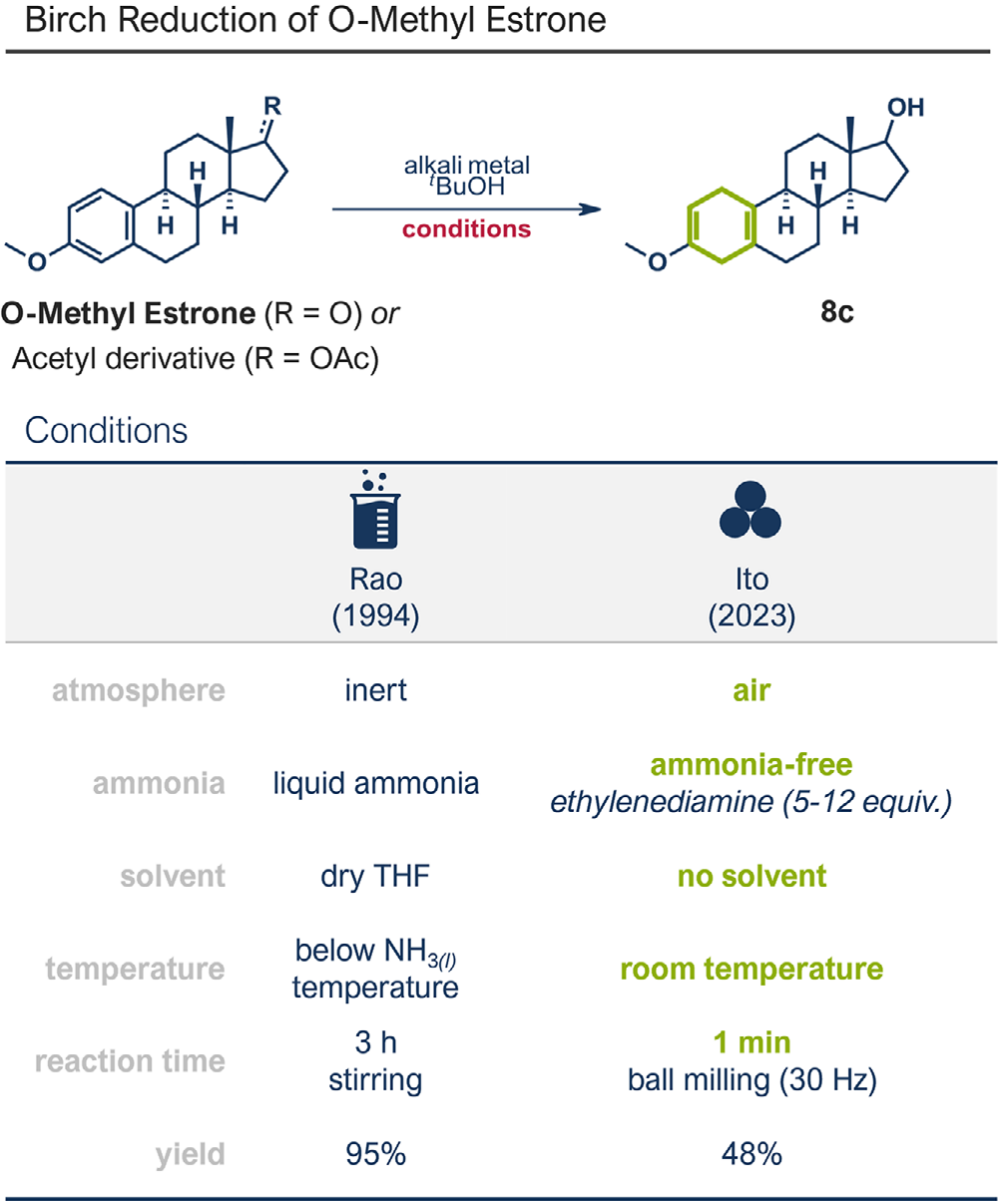
Comparison of the Birch reduction of an estrone derivative under solution‐based^[^ ^197^ ^]^ and mechanochemical conditions.^[^ ^38^ ^]^

This advantage becomes particularly evident when directly comparing mechanochemical and classical solution‐based conditions for specific reactions. A compelling example is the Wittig olefination of the C17‐ketone moiety in the steroid hormone epiandrosterone (Figure 24). Under conventional solution‐based conditions, the reaction requires tedious ylide pre‐formation, strict exclusion of moisture and air, and the use of strong, often pyrophoric bases such as *n*‐BuLi.^[^ ^195^, ^196^ ^]^ The process involves low‐temperature ylide formation, slow addition of the carbonyl substrate, and extended reaction times at elevated temperatures. In contrast, the mechanochemical Wittig olefination discussed in this review eliminates the need for ylide preformation and operates entirely without added solvent under air.^[^ ^33^ ^]^ By simply combining PPh_3_MeBr, KO*^t^* Bu, and the steroid substrate in a milling vessel and grinding at high frequencies, the reaction proceeds efficiently. For epiandrosterone, the transformation is complete in just 5 min at 36 Hz, highlighting the operational simplicity and efficiency inherent to mechanochemical synthesis (see Section 2, Figure 9). Another impressive example of mechanochemistry's advantages over classical solution‐based methods for air‐ and moisture‐sensitive reactions is the Birch reduction, demonstrated here on *O*‐methyl estrone as a model substrate (Figure 25). Traditionally, this reaction requires cryogenic conditions to liquefy ammonia and dissolve an alkali metal such as sodium or lithium, generating an extremely reactive electride species that is highly moisture‐sensitive and poses significant safety hazards.^[^ ^197^ ^]^ In contrast, the groundbreaking work by Kubota, Ito, and coworkers demonstrated an ammonia‐free mechanochemical Birch reduction that operates under ambient conditions with a remarkably short reaction time of just 1 min.^[^ ^38^ ^]^ A thin passivation layer on the lithium metal allows for safe handling in air, but upon ball milling, mechanical forces activate the metal, enabling the selective reduction of various aromatic systems. This operationally simple, solvent‐minimized approach not only improves safety and efficiency but also underscores the transformative potential of mechanochemistry in challenging synthetic transformations.

### 7.2 Zero‐Valent Metal Activation via Mechanical Forces

**Figure 26:**
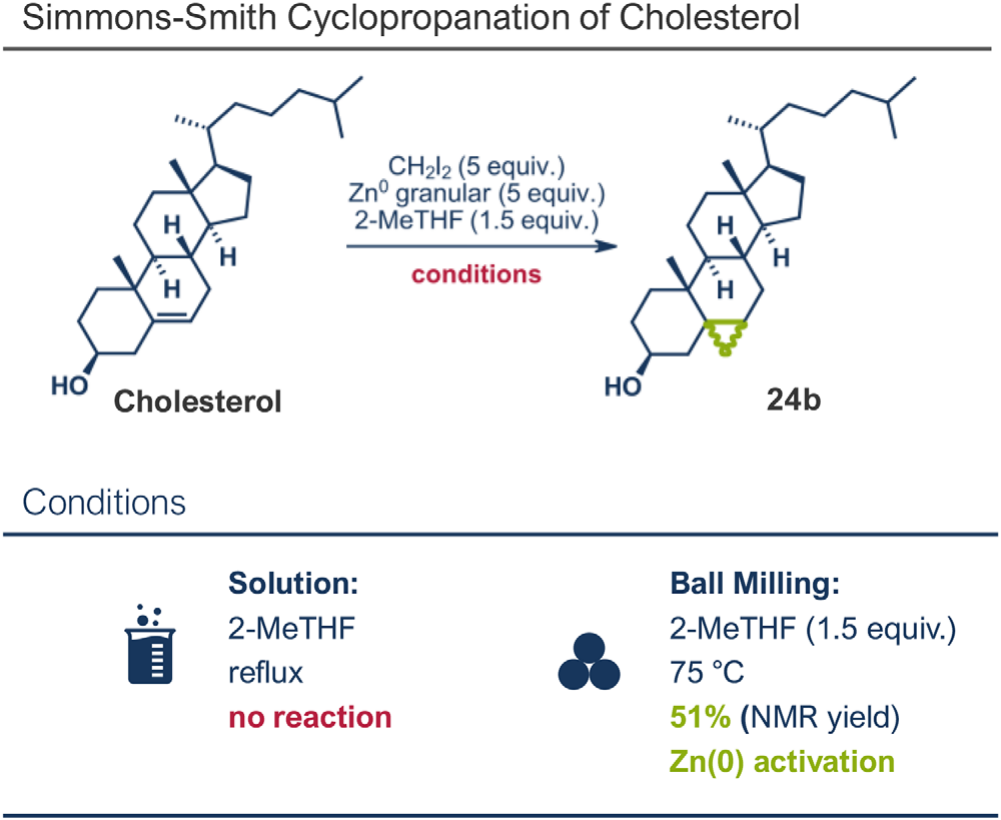
Comparison of solution‐based versus mechanochemical conditions for the Simmons–Smith cyclopropanation of cholesterol by Browne.^[^ ^36^ ^]^

Mechanochemical activation is not limited to lithium; other zero‐valent metals can also be efficiently activated by ball milling, often outperforming traditional solution‐based methods. This enhanced surface activation can enable late‐stage modifications that were previously unsuccessful in solution. One such example was demonstrated by Browne and co‐workers, who achieved a Simmons–Smith cyclopropanation of cholesterol – unattainable via solution‐based methods – through mechanically induced zero‐valent zinc activation (Figure 26).^[^ ^36^ ^]^ Beyond zinc and the previously discussed lithium activation, other metals, such as magnesium, can also be activated through ball milling. This enables their participation in air‐stable Grignard reactions^[^ ^95^ ^]^ or radical‐mediated transformations, as exemplified by Yu and coworkers for the latter (see Section 2, Figure 7).^[^ ^27^ ^]^

### 7.3 Eliminating Solvent‐Related Limitations in Radical and High‐Temperature Reactions

Solution‐based radical reactions often require solvents that can tolerate highly reactive radical species. Common choices include perfluorinated solvents (e.g., hexafluoro isopropanol, HFIP), (chloro‐)benzene, DMF, or DMSO.^[^ ^198^, ^199^ ^]^ However, due to their environmental impact and toxicity concerns, eliminating solvents in these reactions presents an attractive alternative. Without a solvent, there is also no risk of solvent degradation or solvent‐induced by‐product formation, making the solvent‐free approach not only valuable for fundamental research but also highly relevant to the pharmaceutical industry, where solvent costs, environmental impact, and side‐product formation require careful consideration. The studies by Yu and coworkers on Minisci C─H alkylation (see Section 2, Figure 7) and Ito's team on radical trifluoromethylation (see Section 2, Figure 3) highlight this advantage, demonstrating that mechanochemically driven radical reactions can be effectively applied for the late‐stage modification of bioactive compounds.^[^ ^27^, ^29^ ^]^

Beyond minimizing undesired by‐products, the absence of solvents entirely removes hazards associated with flammable or volatile solvents, reducing risks of combustion or explosion – especially critical in large‐scale applications. Moreover, in high‐temperature syntheses, identifying a solvent with a sufficiently high boiling point can be challenging, and even when a suitable solvent is available, its removal during product isolation is often highly energy‐intensive. Although these factors may seem negligible at a small scale, they become significant in industrial processes, making solvent‐free approaches promising alternatives.

### 7.4 Overcoming Solubility Challenges in Chemical Transformations

Depending on their mode and site of action, APIs often require specific structural attributes. For instance, hydrophilic or polar regions can be essential for interactions with biological targets, while lipophilic motifs may need to be introduced to enhance membrane permeability.^[^ ^200^, ^201^ ^]^ However, these structural requirements can pose challenges during synthesis. Many organic transformations necessitate apolar solvents, yet drug‐like molecules with high aqueous solubility often exhibit poor solubility in such media, complicating synthetic modifications.^[^ ^202^ ^]^ This mismatch between solubility properties and reaction conditions can severely limit synthetic accessibility and complicate late‐stage manipulations. Many pharmaceuticals, including antibiotics, nucleoside analogues, and peptide‐based drugs, exhibit high hydrophilicity and strong polarity, which can make them poorly soluble in organic solvents commonly used for synthesis. Such solubility challenges may restrict the efficiency of key transformations or necessitate additional solubility‐enhancing strategies. For example, the synthesis and modification of carbohydrate‐containing small‐molecule drugs using classical solution‐based approaches often encounter these limitations.^[^ ^203^, ^204^, ^205^, ^206^, ^207^ ^]^ Unprotected carbohydrates are typically highly hydrophilic, displaying poor solubility in apolar solvents and, in some cases, even in highly polar organic media.^[^ ^208^ ^]^ When coupling an unprotected carbohydrate moiety with a more apolar building block, phase immiscibility may present a significant obstacle, potentially impeding the reaction. A common strategy to address such solubility mismatches is the introduction of lipophilic protecting groups, such as benzyl ethers, to enhance solubility in organic solvents and improve phase compatibility. However, additional protection and deprotection steps reduce overall atom efficiency and can negatively impact the environmental sustainability of a process. In this context, solvent‐free mechanochemical approaches could offer a promising alternative by enabling direct transformations without the need for solubility‐matching solvents.^[^ ^208^, ^209^ ^]^ Although further exploration is needed, such strategies might help expand synthetic access to highly polar and hydrophilic APIs and their analogues while improving the overall green chemistry profile of the process.

## 8 Challenges and Future Perspectives for Mechanochemistry in Drug Synthesis and Discovery

While mechanochemistry offers promising advantages for the fine chemical and pharmaceutical industries, significant challenges and limitations must be addressed before its full potential can be realized. It should not be regarded as a universal solution to all environmental issues in modern chemical manufacturing. However, it represents a valuable addition to the green chemistry toolbox, contributing to more sustainable and environmentally friendly API research and production. Although the theoretical benefits of mechanochemistry in pharmaceutical applications are compelling, its practical implementation and large‐scale integration remain in their early stages. In the following, we critically assess the current challenges preventing mechanochemical methods from competing with well‐established solution‐based protocols in the pharmaceutical industry while also exploring future opportunities to drive this transition forward.

### 8.1 Device Related Challenges in Mechanochemistry

Several types of mechanochemical devices exist (examples see Section 11), each with advantages and limitations depending on specific synthetic demands. Moreover, reactions are typically developed on a laboratory scale in batch mode before transitioning to pilot plants and eventually to industrial‐scale reactors. Unlike solution‐based systems, which have been extensively studied for scale‐up, reactor modifications, and adaptation to flow processes, mechanochemistry—still an emerging technology—lacks universal parameters that enable seamless interconversion among different mechanochemical devices. This is particularly relevant for devices that operate based on fundamentally different mixing and milling concepts, such as ball milling, extrusion, and resonance acoustic mixing (see Section 11, Figure 29).

Although several reports have attempted to compare horizontal mixers with planetary mills^[^ ^210^, ^211^, ^212^ ^]^ or provide guidance on transitioning from ball milling to RAM^[^ ^84^ ^]^ or extruders,^[^ ^213^ ^]^ there is still an urgent need for standardized parameters and protocols to facilitate smooth transitions between different systems and enable reliable scale‐up of mechanochemical reactions. One potential approach is to quantify the cumulative energy input into the system, which could serve as a universal metric: in theory, if two systems deliver the same cumulative energy—whether primarily through impact forces in a horizontal ball mill or through friction and shear forces in an extruder—the reaction outcome (yield, conversion, and selectivity) should remain comparable.^[^ ^212^ ^]^ However, what appears conceptually straightforward is highly challenging in practice. Precisely identifying and quantifying all contributing parameters for each mechanochemical device to accurately determine cumulative energy remains an open challenge requiring further investigation.^[^ ^214^ ^]^

Energy input, particularly additional thermal energy and efficient heat dissipation, presents another challenge in solvent‐free mechanochemical reactions. In solution‐based batch or flow systems, efficient stirring or a sufficiently high surface area enables effective heat exchange. Additionally, heat transfer in liquid‐phase reactions benefits from convection and advection, where the bulk movement of the solvent facilitates more uniform temperature distribution. In contrast, mechanochemical reactions, typically conducted under solvent‐free or solvent‐minimized conditions, lack this advantage, relying primarily on conduction through solid reactants and milling media. As a result, heat dissipation is often less efficient, posing a challenge for temperature‐sensitive transformations. Although heating or cooling the outer walls of milling vessels or extruders may be sufficient for small‐scale reactions, scaling up thermally accelerated mechanochemical processes requires precise reactor design. A particularly promising technology is the use of extruders – primarily employed in the polymer industry – equipped with a heatable screw. Unlike external reactor wall heating, these systems enable direct heat transfer to the material as it moves through the extruder, ensuring more efficient and uniform thermal control. Although, to the best of our knowledge, such devices have not yet been employed for thermal mechanochemical reactions, we think that this technology holds immense promise for future applications in large‐scale organic synthesis.

Mechanochemistry not only faces large‐scale integration challenges in the pharmaceutical industry but also requires innovation at the other end of the spectrum – high‐throughput small‐scale synthesis. Currently, the only protocol approaching an industry‐attractive level of multireaction setup is the work by Rueping and coworkers, who utilized a 96‐well plate format for rapid reaction parameter screening in RAM.^[^ ^45^ ^]^ No comparable high‐throughput screening devices exist for ball milling systems yet; the largest capacity reported so far is a 12‐vial holder for 2 mL containers in a planetary mill.^[^ ^215^ ^]^ Developing a multivessel holder for ball milling would be a significant engineering milestone, but further challenges remain, particularly in achieving fully automated reaction preparation. Unlike liquid handling in robotic platforms, the precise and reproducible addition of solid reagents to multiple reaction vessels is more complex. A potential solution could involve dissolving reagents in a solvent to facilitate dosing, followed by evaporation to regenerate solid‐phase reactants. However, this approach must carefully prevent unintended reactions during the solution mixing stage. Notably, Rueping's team demonstrated that reaction conditions identified in their high‐throughput RAM setup could be successfully transferred to ball milling reactors.^[^ ^45^ ^]^ This suggests that optimizing reaction parameters in a 96‐well plate RAM system could still provide valuable insights transferable to other mechanochemical platforms, such as ball mills or extruders. These promising results set an important starting point for further advancements in mechanochemical high‐throughput experimentation.

### 8.2 Synthetic Challenges in Mechanochemistry

Apart from the setup‐related challenges mechanochemistry faces in its implementation within the pharmaceutical industry, certain synthetic challenges need to be addressed. This review primarily explores mechanochemistry's potential in the late‐stage modification of existing APIs, emphasizing pharmaceutically relevant protocols that have not yet been comprehensively examined in this context. The focus is largely on strategies that enable slight structural modifications of small‐molecule drugs. However, pharmaceutical research is increasingly shifting toward high molecular weight compounds, such as peptides and their conjugates, highlighting the need for further investigation into the applicability of mechanochemical methods in this area.^[^ ^216^, ^217^ ^]^ Although recent studies have demonstrated the feasibility of mechanochemical small‐peptide synthesis, comprehensive investigations into the stability of long amino acid chains and the modification of existing peptides under these conditions are still lacking, opening a promising field for future research.^[^ ^218^, ^219^, ^220^ ^]^

**Figure 27:**
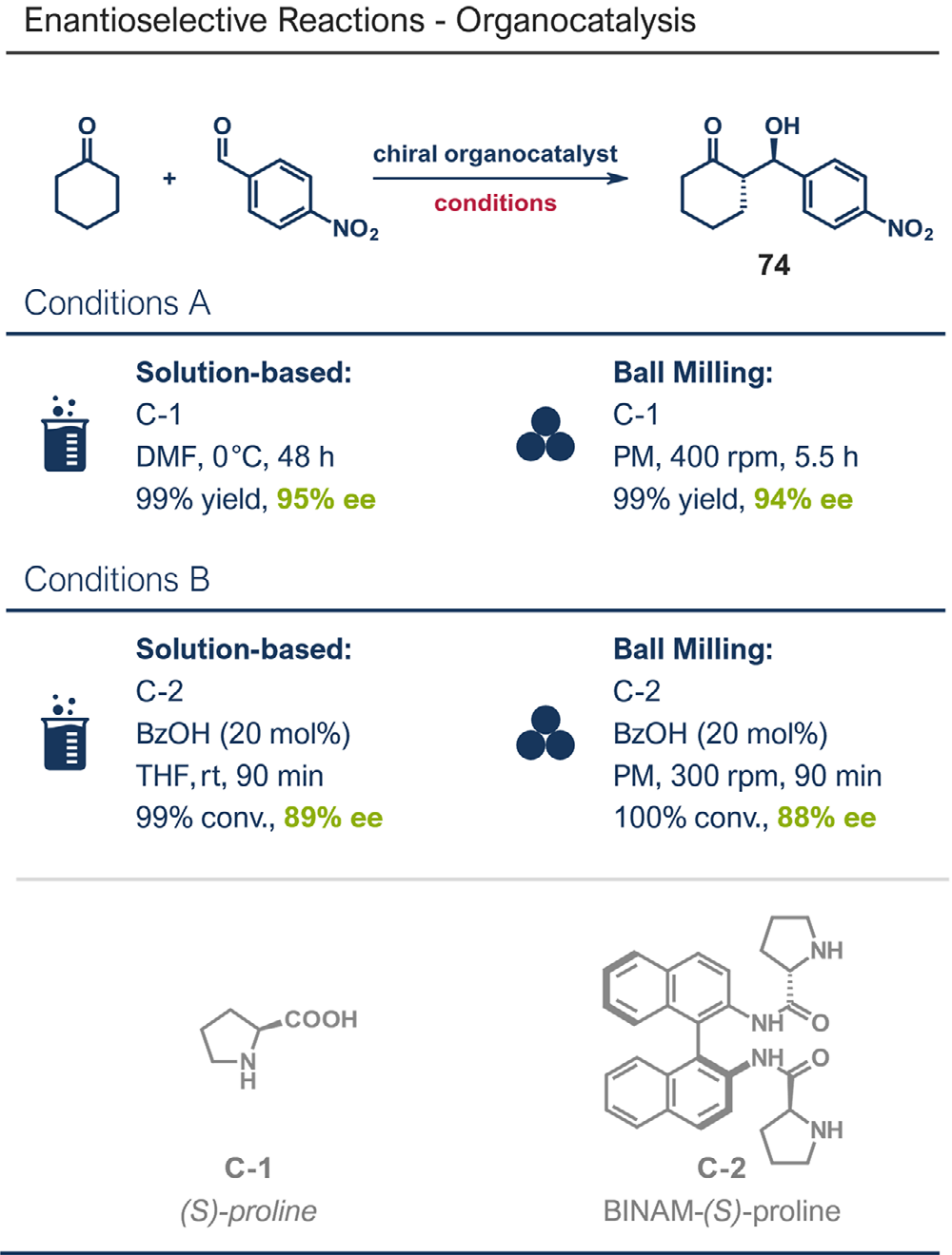
Comparison of enantioselectivity in the aldol reaction under solution‐based and mechanochemical conditions using proline derived organocatalysts.^224^

Furthermore, enantioselective reactions under mechanochemical conditions remain underexplored compared to their well‐established solution‐based counterparts. The significance of enantiopurity in pharmaceuticals is well‐documented – either due to one enantiomer exhibiting harmful effects or because administering the enantiopure form enhances therapeutic efficacy. Thus, for mechanochemistry to gain further traction in drug synthesis, it is essential to develop enantioselective transformations compatible with structurally complex molecules like APIs. So far, most enantioselective mechanochemical reactions have relied on organocatalysis, particularly for selective modifications at the α‐position of carbonyl compounds using chiral secondary or tertiary amines.^[^ ^221^, ^222^, ^223^ ^]^ A review by Browne and colleagues systematically compared the enantiopurity achieved in mechanochemical versus solution‐based organocatalytic reactions, offering valuable insights into their relative efficiency and selectivity.^[^ ^224^ ^]^ A notable example herein is an aldol reaction under ball‐milling conditions catalyzed by *(S)*‐proline (**C‐1**) (Figure 27). Bolm and colleagues demonstrated that cyclohexanone and *p*‐nitrobenzaldehyde reacted efficiently in 5.5 h, yielding the enantioenriched product in 94% *ee* – comparable to the 95% *ee* obtained under solution‐based conditions but with a significantly shorter reaction time (48 h).^[^ ^224^, ^225^, ^226^ ^]^ Similarly, using a 2,2′‐bis‐(diphenylphosphinoamino)‐1,1′‐binaphthyl (BINAM)‐*(S)*‐proline catalyst (**C‐2**), the aldol reaction achieved comparable yields and enantioselectivities in both mechanochemical and solution‐based approaches.^[^ ^227^ ^]^ These studies highlight the potential of organocatalysts to induce high enantioselectivity under mechanochemical conditions.

**Figure 28:**
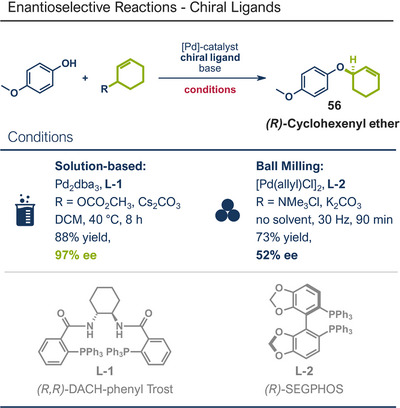
Comparison of ligand‐induced enantioselectivity in the palladium‐catalyzed Tsuji–Trost allylation under solution‐based^[^ ^228^ ^]^ versus mechanochemical^[^ ^30^ ^]^ conditions.

On the other hand, chiral ligand‐induced enantioselectivity in transition metal‐catalyzed coupling reactions under mechanochemical conditions remains largely unexplored. Given their fundamental role in organic synthesis and proven success in solution‐phase enantioselective transformations, further investigation is essential. One example, previously discussed in this review, is the ligand‐induced enantioselective palladium‐catalyzed Tsuji–Trost allylation reported by Templ and Schnürch, which utilized *(R)*‐5,5′‐bis(diphenylphosphino)‐4,4′‐bi‐1,3‐benzodioxole (*(R)*‐SEGPHOS) as a chiral ligand (see Section 4, Figure 16).^[^ ^30^ ^]^ However, a comparison with a solution‐based approach employing an *(R,R)*‐DACH‐phenyl Trost‐ligand shows a significant difference in enantioselectivity, yielding the *(R)‐*cyclohexenyl ether product **56** with 97% *ee* in solution versus 52% *ee* under mechanochemical conditions (Figure 28).^[^ ^228^ ^]^ At present, there is insufficient research to determine whether chiral ligands are inherently less effective under mechanochemical conditions or if reaction parameters require further optimization. We believe that with systematic studies and refined methodologies, mechanochemical enantioselective reactions could become as efficient as their solution‐phase counterparts.

At this stage, the greatest potential for mechanochemistry in the pharmaceutical industry likely lies in its integration into API synthesis, either as an adapted step within an existing solution‐based process or as part of a purposefully designed mechanochemical route. Although its role in high‐throughput experimentation remains an area for further development, mechanochemistry has already demonstrated its ability to minimize solvent use during synthesis. However, complete solvent elimination in industrial API production is unlikely, as solvents are still required for product purification and reactor cleaning. Even so, reducing solvent consumption in selected synthetic steps can significantly enhance the overall environmental sustainability of a process, particularly by avoiding hazardous solvents. Furthermore, as previously discussed, solvent reduction is only one of the many benefits that mechanochemistry offers to pharmaceutical manufacturing.

## 9 Summary and Outlook

Mechanochemistry, as a solvent‐free approach to chemical synthesis, has demonstrated its potential to advance late‐stage functionalization of pharmaceutically active compounds. By addressing key challenges in traditional synthetic methodology – such as environmental sustainability, operational simplicity, and energy efficiency – mechanochemistry has emerged as a green alternative for modifying complex molecular scaffolds. The examples discussed in this review highlight the power of mechanochemistry's application in modifying APIs to enhance their pharmacological properties or to explore structure–activity relationships. Despite these advances, the field is still in its infancy, and significant challenges remain. Similar to traditional solution‐based protocols, mechanochemical methods often lack generality, selectivity, or broad functional group tolerance, an issue of high importance in late‐stage functionalization, where the complexity and functional group diversity of APIs demand robust and highly selective methodologies. Expanding the scope of mechanochemical reactions to include a broader range of bioactive compounds is therefore critical.

Another key challenge lies in scalability. Although small‐scale reactions in laboratory settings have proven successful, the translation of mechanochemical methods to industrial‐scale processes is often not straightforward. Issues such as rheology, heat dissipation, material stress, and reproducibility must be addressed to exploit the full potential of mechanochemistry in pharmaceutical manufacturing. Resonance acoustic mixing and twin‐screw extrusion have shown promise as scalable alternatives to traditional ball milling, but still further optimization and validation is necessary for their adoption.

Furthermore, while the environmental benefits of mechanochemistry are widely touted, critical life‐cycle assessments of these methods are crucial. The energy consumption of ball mills, the environmental impact of milling media, and the use of auxiliary reagents need to be critically evaluated to substantiate claims of sustainability. Hereby, it is essential to consider the broader context of pharmaceutical manufacturing, as transitioning an existing production process to mechanochemistry may require substantial reconstruction of infrastructure, which itself can be energy‐intensive and environmentally impactful. Another limitation is the underexplored field of enantioselective and asymmetric reactions in mechanochemistry. Considering that exactly these asymmetric transformations are often crucial in pharmaceutical synthesis, further research on boosting enantioselectivity in solvent‐free and mechanochemical conditions is key. Looking ahead, interdisciplinary collaboration of scientists and fostering partnerships between academia and industry will be essential for translating laboratory scale mechanochemical methods into industrially viable processes.

Despite these challenges, the potential of mechanochemistry to reshape late‐stage modification and API synthesis becomes obvious. The examples highlighted in this review underscore its ability to meet future needs in pharmaceutical chemistry. The comprehensive tabular summary below, detailing APIs utilized in mechanochemical late‐stage modifications and their corresponding transformations, is designed to be a valuable source for researchers aiming to test or develop new protocols (Table 1). We hope that this review fosters innovative research and development in the field.

## 10 Tabular Overview of APIs discussed

The following table provides a comprehensive summary of all APIs discussed in this review, organized alphabetically by the name of the original or marketed API (Table 1). For each compound, the corresponding mechanochemical late‐stage modifications and any necessary prefunctionalizations are provided. This detailed overview is designed to assist and inspire researchers in selecting pharmaceutically active compounds for testing the synthetic utility of novel and existing (mechano)chemical protocols. We hope this resource contributes to advancing the field of mechanochemical late‐stage functionalizations.

## 11 Mechanochemical Devices

**Figure 29:**
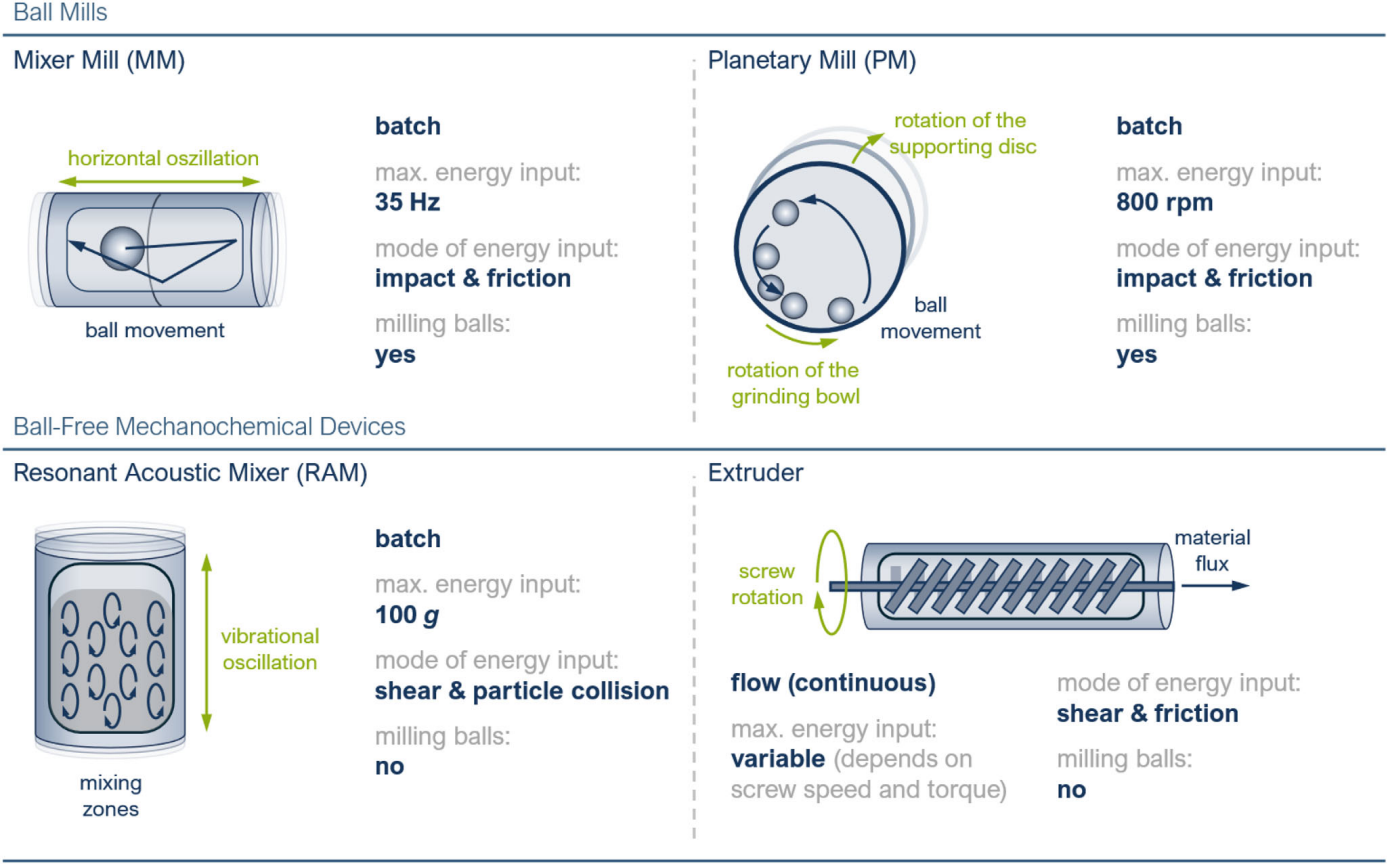
Schematic representation of the mechanochemical devices used in the discussed syntheses, highlighting their fundamental operating principles, mode of operation (batch/continuous), typical maximum energy input, and predominant energy transfer mechanisms.

Mechanochemical transformations can be performed using a range of devices, each differing in energy input mechanisms and operating principles (for examples thereof discussed within this review see Figure 29). The most commonly used instruments include mixer mills, planetary mills, resonant acoustic mixers (RAM), and extruders, each offering distinct advantages in terms of mechanical forces, scalability, and reaction suitability.

**Mixer mills** operate in batch mode, utilizing high‐frequency oscillatory motion to transfer energy through impact and friction. The reaction vessel, typically loaded with milling balls, moves rapidly in a back‐and‐forth motion, generating intense collisions that drive chemical transformations. These devices are widely employed for small‐scale reactions and are valued for their ease of operation.

**Planetary mills** also function in batch mode but provide significantly higher energy input than mixer mills. Their grinding jars rotate around their own axis while simultaneously orbiting a central axis, generating strong centrifugal and shear forces. This dual motion enhances reaction efficiency and is particularly advantageous for processes requiring high energy input.

**Resonant acoustic mixers (RAM)** offer a distinct approach by employing low‐frequency acoustic vibrations to induce particle movement and mixing. Unlike ball mills, RAM devices do not rely on milling balls but instead use acoustic energy to create shear and collision forces within the sample. This method allows for gentler mixing, making it particularly suitable for processing sensitive materials and enabling high‐throughput reaction screening.

**Extruders** provide a continuous, flow‐compatible mechanochemical platform, where reactants experience mechanical shear and compression along a rotating screw. This setup enables scalable synthesis while improving heat dissipation, addressing some of the limitations of batch‐based milling. The ability to precisely control shear intensity makes extruders especially promising for industrial applications, including polymer processing and organic synthesis.

## Conflict of Interests

The authors declare no conflict of interest.

## Data Availability

Data sharing is not applicable to this article as no new data were created or analyzed in this study.
